# Investigation of C1-complex regions reveals new *C1Q* variants associated with protection from systemic lupus erythematosus, and affect its transcript abundance

**DOI:** 10.1038/s41598-018-26380-x

**Published:** 2018-05-23

**Authors:** Jianping Guo, Yanyan Gao, Yuxuan Wang, Yundong Zou, Yan Du, Cainan Luo, Yamei Shi, Yue Yang, Xinyu Wu, Yin Su, Lijun Wu, Shi Chen, Zhanguo Li

**Affiliations:** 1 Department of Rheumatology and Immunology, Peking University People’s Hospital, Beijing, China; 2 Department of Neurology, Children’s Hospital Affiliated to Capital Institute of Pediatrics, Beijing, China; 3 Department of Rheumatology and Immunology, The People’s Hospital of Xinjiang Uygur Autonomous Region, Urumqi, China

## Abstract

Although rare variant *C1Q* deficiency was identified as causative risk for systemic lupus erythematosus (SLE), there are limited and inconsistent reports regarding the common polymorphisms of *C1Q* genes in SLE susceptibility. Furthermore, there are no reports concerning polymorphisms of *C1S*, *C1R*, and *C1RL* and whether they confer susceptibility to SLE. We therefore evaluated 22 SNPs across six C1-complex genes in two independent case-control cohorts, and identified four novel SNPs that confer protection from SLE. The four SNPs are all located in *C1Q*. Particularly, the variant rs653286 displayed an independent reduced risk on SLE susceptibility (OR 0.75, *P* = 2.16 × 10^−3^) and anti-dsDNA antibodies (OR 0.68, *P* = 0.024). By bioinformatics analysis, SNPs rs653286 and rs291985 displayed striking *cis*-eQTL effects on *C1Q* genes expression. Individuals homozygous for the ‘protective’ allele at four SNPs had significantly higher levels of serum C1q (rs680123–rs682658: *P* = 0.0022; rs653286–rs291985: *P* = 0.0076). To our knowledge, this is the first study to demonstrate that only *C1Q* polymorphisms are associated with SLE. The *C1Q* SNP rs653286 confers an independent protective effect on SLE susceptibility and affects transcript abundance.

## Introduction

Systemic lupus erythematosus (SLE) is a chronic and systemic autoimmune disease, clinically characterized by heterogeneous manifestations and production of multiple autoantibodies. The exact etiology of SLE is unknown, but it is generally acknowledged that the disease evolves a complex interplay between multiple environmental and genetic factors.

Complement cascade molecules are the key component in innate immune activation and play an important role in SLE pathogenesis. The C1-complex, composed of three molecules C1q, C1r, and C1s, is the first component in activation of classical complement pathway. The C1q molecule is composed of three similar but different types of chains, namely A-, B-, and C-chains, which are encoded by three distinct genes, i.e. *C1QA*, *C1QB*, and *C1QC*. The genes are arranged tandemly in order of A-C-B on a 24-kb stretch of DNA on human chromosome 1p36^1^. Human *C1S* and *C1R* are closely located at a distance of approximately 9.5 kb on chromosome 12p13. Both *C1S* (10.5 kb) and *C1R* (11 kb) contain 12 exons, and are highly homologous in domain structure and function^2,3^. Furthermore, the complement C1r-like protein (*C1RL*) is a recently discovered homologue of *C1R*, located 2 kb centromeric to *C1R*^4^.

Although *C1Q* and *C1S* deficiencies were identified as causative genetic risks for SLE (with a disease incidence of 93% and 68%, respectively), such deficiencies are very rare. To date fewer than 90 individuals with C1 deficiencies have been reported worldwide: approximately 70 cases with *C1Q* deficiency and 19 cases with *C1S* deficiency^5,6^. There are limited and inconsistent reports regarding the common polymorphisms in *C1Q* cluster in relation to SLE susceptibility^7–12^. One publication evaluated the association of *C1Q* polymorphisms with SLE in Han population, using a candidate gene approach and focusing only on *C1QA*^10^. There are no reports on genetic association of *C1S*, *C1R*, *and C1RL* polymorphisms and susceptibility to SLE. We therefore undertook the current study to investigate the possible genetic association(s) between the six C1genes (*C1QA*, *C1QC*, *C1QB*, *C1S*, *C1R*, and *C1RL*) and SLE susceptibility or clinical/serologic features. We report that *C1Q* polymorphisms, but not polymorphisms in *C1S* and *C1R*, are protective against SLE susceptibility and *affect C1Q* transcript abundance.

## Results

A total of 22 SNPs were selected for the discovery screening. These included 18 tag-SNPs spanning *C1QA*, *C1QC*, *C1QB*, *C1S*, *C1R*, and *C1RL* loci and additional 4 SNPs known to be associated with SLE or related symptoms^9^ (Table 1). All SNPs were in Hardy-Weinberg equilibrium (HWE) in both patients and controls (*P* > 0.05, data not shown). In control groups, the allele frequencies of SNPs were similar to the data from HapMap CHB (shown in Tables 1, 2 and Supplementary Table S1).

**Table 1:** Information of SNPs from HapMap CHB database.

Chr.	Gene	SNP^a^	Position (Mb)	Alleles^b^	MAF	Functional alteration	HWpval
1	C1QA	rs680123	22635031	C/T	0.329	nearGene-5′	0.2733
1	C1QA	**rs12033074**	22640116	G/C	0.433	nearGene-3′	0.1502
1	C1QC	**rs4655085**	22641932	A/G	0.151	nearGene-5′	0.5839
1	C1QC	rs682658	22642322	G/T	0.329	nearGene-5′	0.2733
1	C1QC	rs655903	22644526	G/A	0.256	Intron	1.0000
1	C1QC	**rs672693**	22644953	A/G	0.369	Intron	0.6547
1	C1QC	rs653286	22645091	T/C	0.300	Intron	0.6547
1	C1QB	rs12754182	22651775	T/C	0.211	nearGene-5′	0.2733
1	C1QB	rs17433222	22652153	A/G	0.150	nearGene-5′	0.5839
1	C1QB	rs913243	22653494	T/G	0.386	intron	0.3428
1	C1QB	rs291985	22654446	T/G	0.356	intron	0.4027
1	C1QB	**rs629409**	22660245	A/G	0.439	intron	0.2683
12	C1S	rs7962629	7059466	G/A	0.073	nearGene-5′	1.0000
12	C1S	rs12146727	7063032	A/G	0.073	missense	1.0000
12	C1S	rs7183	7070715	T/G	0.073	UTR-3′	1.0000
12	C1R	rs3813728	7088974	T/C	0.085	missense	1.0000
12	C1R	rs7135975	7090972	G/A	0.183	intron	0.5271
12	C1RL	R s7709	7094764	C/A	0.207	UTR-3′	1.0000
12	C1RL	rs3782928	7096079	A/G	0.159	UTR-3′	0.2733
12	C1RL	rs3742089	7097002	G/A	0.354	missense	0.5839
12	C1RL	rs12304029	7098579	C/G	0.364	intron	1.0000
12	C1RL	rs3742088	7102073	G/T	0.171	cds-synon	1.0000

CHB: Chinese Han in Beijing; Chr: chromosome; SNPs: single nucleotide polymorphisms; MAF: minor allele frequency; UTR: untranslated region; missense: missense mutation; cds-synon: coding region variant-synonymous mutation; HWpval: *p*-value for Hardy-Weinberg equilibrium;

^a^SNPs in bold: previously known to be associated with SLE or related symptoms;

^b^Minor allele/Major allele.

**Table 2:** Associations between 4 SNPs in *C1Q* genes with SLE susceptibility, logistic regression adjusting for age and gender.

Gene	SNP	MAF* Cases/Cons	Models	Stage I		Stage II		Combined			
				OR (95%CI)	*p*-value	OR (95%CI)	*p*-value	OR (95% CI)	*p*-value	*I*^2^ (%)	*p*-value (Het)
C1QA	rs680123	0.350/0.369	Allelic (C/T)	0.85 (0.69–1.06)	0.143	0.93 (0.78–1.10)	0.394	0.90 (0.79–1.03)	0.116	0	0.55
			Recessive (CC/CT + TT)	1.05 (0.69–1.59)	0.822	1.04 (0.74–1.45)	0.833	1.04 (0.80–1.35)	0.788	0	0.39
			Dominant (CC + CT/TT)	0.72 (0.54–0.97)	**0.028**	0.85 (0.67–1.08)	0.186	0.80 (0.66–0.96)	**0.016**	0	0.46
C1QC	rs682658	0.351/0.372	Allele (G/T)	0.87 (0.70–1.07)	0.187	0.94 (0.79–1.12)	0.489	0.91 (0.80–1.04)	0.187	0	0.57
			Recessive (GG/GT + TT)	1.10 (0.73–1.66)	0.652	1.05 (0.75–1.48)	0.772	1.07 (0.82–1.39)	0.631	0	0.38
			Dominant (GG + GT/TT)	0.72 (0.54–0.97)	**0.032**	0.87 (0.68–1.10)	0.240	0.81 (0.67–0.97)	**0.024**	0	0.47
C1QC	rs653286	0.332/0.368	Allele (T/C)	0.83 (0.67–1.03)	0.091	0.88 (0.73–1.04)	0.138	0.86 (0.75–0.98)	**0.024**	0	0.44
			Recessive (TT/TC + CC)	1.03 (0.65–1.62)	0.905	0.99 (0.70–1.40)	0.960	0.99 (0.76–1.31)	0.993	0	0.51
			Dominant (TT + TC/CC)	0.70 (0.52–0.94)	**0.018**	0.78 (0.62–0.99)	**0.045**	0.75 (0.62–0.90)	**2.45** × **10**^**−3**^	0	0.39
C1QB	rs291985	0.335/0.368	Allele (T/G)	0.87 (0.71–1.08)	0.218	0.86 (0.73–1.03)	0.098	0.87 (0.76–0.99)	**0.035**	0	0.34
			Recessive (TT/TG + GG)	1.08 (0.69–1.70)	0.740	1.01 (0.72–1.42)	0.940	1.03 (0.79–1.35)	0.828	0	0.33
			Dominant (TT + TG/GG)	0.75 (0.56–1.01)	**0.059**	0.75 (0.59–0.95)	**0.019**	0.76 (0.63–0.91)	**2.97** × **10**^**−3**^	0	0.67

SLE: systemic lupus erythematosus; SNPs: single nucleotide polymorphisms; OR (95% CI): odds ratio (95% confidence interval);

*Minor allele frequencies in combined cohort (cases/controls).

*p*-value (Het): *p*-value for heterogeneity, *I*^2^: heterogeneity statistic.

### Discovery screening of C1-complex genomic regions reveals *C1Q* polymorphisms associated with SLE susceptibility

We first sought to investigate any potential association(s) between the selected SNPs resided in C1-complex and SLE susceptibility in the discovery cohort. As shown in Tables 2 and S2, although at the allele level no association was observed, at the genotype level we found three SNPs that conferred nominal protective effects against SLE (dominant model: *C1QA* rs680123: OR 0.72, *P* = 0.028, *q* = 0.235; *C1QC* rs682658: OR 0.72, *P* = 0.032, *q* = 0.235; and *C1QC* rs653286: OR 0.70, *P* = 0.018, *q* = 0.235, respectively). A suggestive reduced risk effect was also found for *C1QB* rs291985 (dominant model: OR = 0.75, *P* = 0.059, *q* = 0.325). Notably, the four SNPs are all resided in *C1Q* genes. No potential association was observed for other polymorphisms outside *C1Q* in SLE susceptibility (Supplementary Table S1).

### Replication study and joint analysis confirms the reduced risk of the four *C1Q* polymorphisms, but only SNP rs653286 has an independent protective effect on SLE susceptibility

To confirm the possible associations that we observed in the discovery cohort, the top 4 SNPs were replicated in an independent case-control cohort. Joint analysis was then performed by combining results from discovery and replication cohorts.

As shown in Tables 2 and S2, in the replication panel, both rs653286 and rs291985 showed consistent protection from SLE (dominant model: rs653286: OR 0.78, *p* = 0.045, *q* = 0.090; rs291985: OR 0.75, *P* = 0.019, *q* = 0.076, respectively). A trend toward a protective effect was also observed for rs680123 and rs682658, although this negative association did not reach statistical significance.

Joint analysis of discovery and replication panels showed that all the four SNPs conferred protective effects on SLE susceptibility (Tables 2 and S2). In particular, the variants rs653286 and rs291985 showed more pronounced protective effects both at allele (rs653286: OR_combined_ = 0.86, *P* = 0.024, *q* = 0.070; rs291985: OR_combined_ = 0.87, *P* = 0.035, *q* = 0.070, respectively) and genotype levels (dominant model: rs653286: OR_combined_ = 0.75, *P* = 2.45 × 10^−3^, *q* = 5.94 × 10^−3^; rs291985: OR_combined_ = 0.76, *P* = 2.97 × 10^−3^, *q* = 5.94 × 10^−3^, respectively) than the variants rs680123 and rs682658 did (Tables 2 and S2). There was no evidence for heterogeneity between two sample sets in the four variants (allele model: *P*_*het*_ ≥ 0.50, *I*^2^ = 0%; genotype model: *P*_*het*_ ≥ 0.33, *I*^2^ = 0%, shown in Table 2).

Subsequent haplotype analysis showed consistent results supporting the protective effects of the four C1Q polymorphisms on SLE susceptibility (Supplementary Results, Table S3). The variants rs680123–rs682658, and rs653286–rs291985 are almost in perfect LD (r^2^ = 0.96/0.97 in controls/cases, respectively) (Supplementary Figure S1).

To test the independence of the four SNPs, a stepwise (forward conditional) logistic regression analysis was performed in the combined cohort. As shown in Table 3, only rs653286 was independently associated with the disease at both allelic (*P* = 0.022) and genotypic (dominant model: *P* = 2.16 × 10^−3^) levels. Subsequent additions of rs291985, rs682658 and rs680123 did not show significant association with SLE susceptibility.

**Table 3:** Independent effects among the identified 4 SNPs in C1Q genes, stepwise logistic regression (forward conditional).

Models	B	*P*-value	OR (95% CI)
Allelic (m/M)			
rs653286	−0.159	0.022	0.85 (0.75–0.98)
rs291985	—	0.219	—
rs682658	—	0.743	—
rs680123	—	0.913	—
Recessive (mm/mM + MM)			
rs653286	—	0.891	—
rs291985	—	0.930	—
rs680123	—	0.798	—
rs682658	—	0.556	—
Dominant (mm + mM/MM)			
rs653286	−0.291	2.16 × 10^−3^	0.75 (0.62–0.90)
rs291985	—	0.564	—
rs680123	—	0.444	—
rs682658	—	0.623	—

B: logistic regression beta coefficients; OR (95% CI): odds ratio (95% confidence interval); m: minor allele; M: major allele.

To investigate whether the independent protective SNP rs653286 predispose to any particular disease manifestation(s), we assessed the association(s) between SNP rs653286 and clinical/serologic features in a case-only cohort. Following stratification, we found SNP rs653286 was significantly associated with anti-dsDNA antibodies (dominant model: OR 0.68, *P* = 0.024). However, no association was observed for other SLE manifestations (Table S4). This finding might be explained by the limited statistical power of this analysis.

### Bioinformatics annotations support potential regulatory function(s) of the four *C1Q* variants

**Figure 1:**
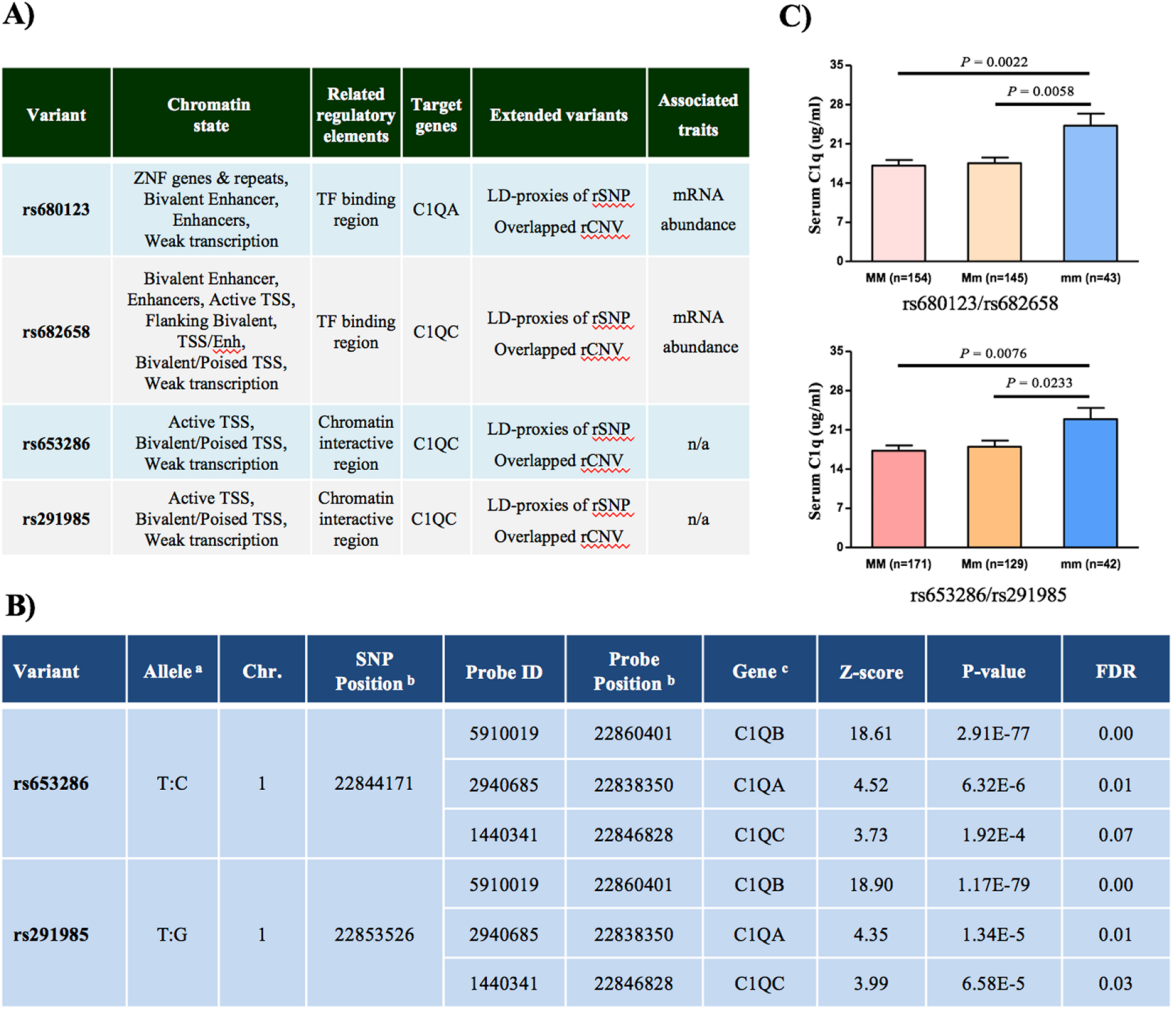
Bioinformatics annotations and genotype-dependent expression analysis supporting the functional role of *C1Q* rs653286 and rs291985. (**A**) The four variants rs680123, rs682658, rs653286, and rs291985 all displayed potential regulatory activities in rVarBase database. (**B**) SNPs rs653286 and rs291985 displayed striking *cis*-eQTL effects on three *C1Q* genes expression (**B**,**A** and **C**) in the Blood eQTL database. ^a^Minor allele/Major allele; ^b^the genomic position cited from Blood eQTL database; ^c^potentially regulated genes by the regulatory variants. (**C**) Genotype-dependent analysis of serum C1q levels. Individuals homozygous for the ‘protective’ allele “m/m” at four SNPs had significantly higher levels of serum C1q expression, compared to individuals homozygous for the major allele “M/M” or heterozygous “M/m”.

As the four variants are localized in either near 5′-UTR or introns, we performed bioinformatics analysis to access their potential regulatory effects. As annotated in rVarBase database, the four variants all displayed potential regulatory activities, such as location within TF (transcription factor) binding sites or chromatin interactive regions, showing LD-proxies with rSNPs or overlapping with rCNVs, regulating TSS (transcription start site) or transcriptional enhancers, and/or association with mRNA abundance, etc. (Fig. 1A). By searching in the RegulomeDB database, the four variants also showed regulatory potential by affecting protein binding, chromatin structure, and histone modifications (Supplementary Table S5). By searching into the Blood eQTL database, we found SNPs rs653286 and rs291985 displayed striking *cis*-eQTL (expression Quantitative Trait Loci) effects on expression of *C1QB* (rs653286: *P* = 2.91 × 10^−77^, FDR = 0.00, Z score = 18.61; rs291985: *P* = 1.17 × 10^−79^, FDR = 0.00, Z score = 18.90), *C1QA* (rs653286: *P* = 6.32 × 10^−6^, FDR = 0.01, Z score = 4.52; rs291985: *P* = 1.34 × 10^−5^, FDR = 0.01, Z score = 4.35), and *C1QC* (rs653286: *P* = 1.92 × 10^−4^, FDR = 0.07, Z score = 3.73; rs291985: *P* = 6.58 × 10^−4^, FDR = 0.03, Z score = 3.99) (Fig. 1B).

### Genotype-dependent expression analyses are further evidence of the ‘protective’ role of the four *C1Q* variants

Supported by the functional annotations, we next sought to determine whether the ‘protective’ alleles at the four SNPs had any impact on serum C1q levels. As SNPs rs680123–rs682658 and rs653286-rs291985 are almost in complete LD (r^2^ = 0.96/0.97 in controls/cases, respectively), we defined individuals homozygous for minor alleles of the four SNPs as “m/m” genotype and individuals homozygous for the major alleles as “M/M” genotype, respectively. As shown in Fig. 1C, in concordance with our association data and *in silico* functional annotations, individuals homozygous for the ‘protective’ allele “m/m” at all four SNPs had significantly higher levels of serum C1q expression, compared to individuals homozygous for the major allele “M/M” (rs680123–rs682658: *P* = 0.0022; rs653286–rs291985: *P* = 0.0076) or heterozygous “M/m”(rs680123–rs682658: *P* = 0.0058; rs653286–rs291985: *P* = 0.0233).

## Discussion

Although the rare deficiencies of *C1Q* and *C1S* have been reported as causative genetic risks for SLE and the common variants resided in *C1Q* have been linked to SLE, there are no reports concerning the polymorphisms of *C1R* and *C1S* in susceptibility to SLE. In present study, we investigated the possible association(s) of the six C1-complex genes, i.e. *C1QA*, *C1QC*, *C1QB*, *C1S*, *C1R*, and a novel human complement-related gene *C1RL*, with SLE susceptibility or its clinical/serologic manifestations. We demonstrate that only *C1Q* but not *C1R*, *C1RL*, and *C1S* polymorphisms, are negatively associated with SLE. The *C1QC* SNP, rs653286, has an independent protective effect on SLE susceptibility. Bioinformatics annotations and genotype-dependent expression analyses further support a potential regulatory role for each of the four *C1Q* variants.

Several studies have linked the common *C1Q* polymorphisms to SLE susceptibility or sub-phenotypes^8,9,12^. Among those, Martens *et al*.^8^ reported that *C1QB* rs631090 was associated with SLE susceptibility, *C1QA* rs292001 and *C1QC* rs294183 was associated with more severe disease in 103 patients with SLE and their first degree relatives of a Caucasian cohort. Though the three SNPs were not tested in present study, they were captured by our candidate SNPs rs629409 (rs631090–rs629409, r^2^ = 0.88), rs680123 (rs292001–rs680123, r^2^ = 1.0), and rs653286 (rs294183–rs653286, r^2^ = 0.84), respectively (shown in Supplementary Figure S2). Among these, rs680123 and rs653286 were shown association with SLE in present study. Interestingly, the two SNPs conferred protective effects in our study. Though rs629409 was in strong LD with rs631090 and was also reported as a risk for SLE^9^, no association is observed between this variant and SLE in the present work. We also genotyped three additional SNPs, rs12033074, rs4655085, and rs672693, known as risk variants for SLE or subphenotypes^9^. However, none of these SNPs demonstrated any association in our study. This finding may indicate that genetic heterogeneity exists among different populations. In addition, *C1QA* rs172378 has been reported to be associated with photosensitivity in lupus patients in African American and Hispanic populations^9^. However, in a previous study that used a candidate gene approach, rs172378 was not shown to have any associations with SLE in a Han Chinese cohort^10^. As the polymorphism rs172378 was not a tagSNP according to HapMap phase III CHB panel, it was not included in our study.

As the four polymorphisms reside in near 5′-UTR or introns, their functional consequence and the mechanism(s) underlying this genetic association remain unclear. However, by *in silico* functional annotations, the four variants all displayed potential regulatory activities. SNPs rs653286 and rs291985 also showed strong *cis*-eQTL effects on *C1Q* gene expression. Furthermore, several studies have suggested that functionally impaired C1q may contribute to SLE pathogenesis^13,14^. Active disease in patients with SLE is often accompanied by low levels of C1q and other classical complement components^15,16^. Restoration of C1q levels by plasma transfusion in C1q-deficient lupus patients resulted in amelioration of the disease^17^. These data are biologically consistent with our findings:, the individuals homozygous for the ‘protective’ allele “m/m” at the four SNPs had significantly higher levels of serum C1q. Interestingly, the ‘protective’ T allele at rs653286 also conferred reduced risk for anti-dsDNA antibodies. Anti-dsDNA antibodies are one of the putative serologi**c** markers for diagnosis of SLE. Levels of circulating anti-dsDNA antibodies fluctuate with disease activity in lupus patients. Furthermore, Yang *et al*. reported that the combination of anti-C1q and anti-dsDNA autoantibodies indicated higher renal disease activity and predicted poor renal outcome in patients with lupus nephritis^18^. Human anti-DNA antibodies could cross-react with C1q and deposit in the kidney in lupus patients^19^. The protective effect of rs653286 T allele on this serologic manifestation may explain, at least in part, its consistent association with increased serum C1q expression in SLE patients.

In summary, to best of our knowledge, this is the first report to investigate the genetic association between the six C1-complex genes and susceptibility to SLE. Our data indicate that only *C1Q* polymorphisms confer susceptibility to SLE. The four novel variants all confer reduced risk against SLE and affect its transcript abundance. The *C1QC* SNP, rs653286, has an independent protective effect on SLE susceptibility. Future association studies in other populations will be required to further confirm our findings. Future functional characterization of these polymorphisms is warranted to fully understand their contributions to SLE pathogenesis.

## Materials and Methods

### Study design and study population

A two-stage case-control study was conducted. Two independent cohorts, including 384 SLE patients and 384 healthy controls (discovery cohort), as well as 507 SLE patients and 645 controls (replication cohort), were enrolled in the study. Patients with SLE satisfied 1982 revised American College of Rheumatology classification criteria for a diagnosis of SLE^20^. Autoantibodies, including anti-nuclear antibodies (ANA), anti-double stranded DNA (dsDNA) antibodies, anti-SSA/SSB antibodies, anti-Smith (Sm) antibodies, anti-cardiolipin antibodies (ACA), and anti-histone antibodies (AHA) were routinely measured and are detailed in the Supplementary Materials and Methods.

The SLE patients in the discovery and replication cohorts were recruited from the Department of Rheumatology at Peking University People’s Hospital and People’s Hospital of Xinjiang Province, respectively. The healthy controls were recruited from Health Care Centers of People’s Hospital. All patients and healthy controls were Han Chinese. The baseline demographic characteristics of patients and healthy controls were summarized in Table 4.

**Table 4:** Demographic characteristic of the study cohorts.

	Stage I (Discovery)	Stage II (Replication)
No. of patients/controls	384/384	507/645
Demographic characteristics		
Female (%)	90.3/89.3	89.4/90.5
Age, mean ± SD years	35.5 ± 12.2/42.0 ± 5.4	37.3 ± 14.2/42.6 ± 11.2
Clinical characteristics		
Age at onset, mean ± SD years	29.0 ± 10.9	32.7 ± 13.2
Disease duration, mean ± SD years	5.4 ± 6.4	5.8 ± 5.7
SLEDAI [median (IQR)]	4 (2–9)	4 (1–6)
Clinical manifestations, n (%)*		
Rash	326 (68.7)	300 (66.7)
Photosensitivity	309 (39.2)	267 (32.6)
Raynaud	308 (34.4)	203(36.0)
Arthritis	343 (65.0)	242 (71.5)
Renal disorder	200 (69.1)	220 (56.8)
Neurological disorder	196 (9.2)	42 (7.1)
Leukopenia	222 (68.0)	181 (40.9)
Thrombopenia	208 (40.9)	180(25.6)
Complement depressed	242 (84.7)	185 (57.3)
Autoantibody positivity, n (%)*		
ANA	350 (92.6)	308 (94.8)
Anti-dsDNA	315 (67.6)	285(58.0)
Anti- SSA	303 (41.6)	281(54.8)
Anti- SSB	277 (13.4)	271(23.6)
Anti- Sm	298 (27.9)	262 (27.9)
ACA	250 (21.2)	224 (17.0)
Anti-RNP	290 (30.3)	256 (35.2)
AHA	198 (20.2)	175 (18.3)

SLEDAI: SLE Disease Activity Index; ANA: antinuclear autoantibodies; Anti-dsDNA: anti-double stranded DNA antibodies; Anti- SSA: anti- SSA antibody; Anti- SSB: anti- SSB antibody; Anti-Sm: anti-Smith antibody; ACA: anti-cardiolipin antibodies; Anti-RNP: anti-ribonucleoprotein antibodies; AHA: anti-histone antibodies. SD: standard deviation; IQR: inter quartile rank.

*“n” indicating the number of cases when the data were available and “%” indicating the proportion of positivity of the manifestations.

The study was approved by Medical Ethics Committee in Peking University People’s Hospital and written informed consent was obtained from all participants. All methods were performed in accordance with the relevant guidelines and regulations.

### SNP selection and genotyping

A total of 22 SNPs were selected for the discovery screening, including 18 tag-SNPs spanning the *C1QA*, *C1QC*, *C1QB*, *C1S*, *C1R*, and *C1RL* loci and an additional 4 SNPs known to be associated with SLE or related symptoms (Table 1). Linkage disequilibrium (LD) tag-SNPs were selected with a threshold of r^2^ < 0.8 and a minor allele frequency (MAF) ≥5% using Haploview v4.2, according to the HapMap phase III Chinese Han Beijing (CHB) panel (http://hapmap.ncbi.nlm.nih.gov/, Figure S2). For the validation analysis, the top 4 SNPs with significant or nominal associations from the discovery cohort were further genotyped in the replication cohort.

All SNPs were genotyped using Sequenom MassArray platform (Sequenom, San Diego, California), and performed at Beijing SequeSci Co., Ltd. Briefly, DNA from study subjects was randomly assigned to the 96 well plates, and genotyping was performed blind to the status of all the samples. Genotyping was repeated in 5% of the samples for validation and quality control. The genotyping error rate was less than 0.1%. Individuals with genotyping success rates less than 90% were excluded from the analyses. Individual SNP markers with more than 10% missing genotypes were also removed from the analyses.

### Quantification of human C1q in serum

A total of 342 genotyped SLE patients were quantified for serum C1q when sera were available. All the serum samples were derived from Peking University People’s Hospital. Human C1q Platinum ELISA kit (BMS2099) was used to measure serum C1q, according to the manufacturer’s instructions (eBioscience, San Diego, CA). In brief, an anti-human C1q monoclonal antibody is coated and adsorbed onto microwells. The absorbance was measured at 450 nm. The concentration of C1q in a serum sample was determined by matching its absorbance with the corresponding C1q concentration in the standard curve. Samples were run in duplicate and analyzed individually. All cases had genotyping data.

### Bioinformatics analysis

The variant’s potential regulatory features were annotated according to RegulomeDB (http://regulome.stanford.edu/) and rVarBase (http://rv.psych.ac.cn/). RegulomeDB is a database that annotates SNPs with known and predicted regulatory features in the non-coding regions of the human genome. Known and predicted regulatory DNA elements include sites of DNAase hypersensitivity, binding sites for transcription factors, and promoter regions that have been biochemically characterized to regulate transcription. The sources of these data include public datasets from GEO, the ENCODE project, and published literature^21^. rVarBase annotates a variant’s regulatory features in following aspects: chromatin state of the region surrounding the variant, regulatory elements overlapped with the variant, and the variant’s potential target genes. rVarBase also provides additional extended annotations for variants, including: LD-proxies of known SNPs, SNP/CNV that are overlapped with or co-localize with the queried variant, and traits (disease and expression quantitative trait) associated with the variant^22^. The blood eQTL data were derived from Blood eQTL browser (http://genenetwork.nl/bloodeqtlbrowser/)^23,24^.

### Statistical analyses

The HWE test was performed for each polymorphism, using Pearson’s goodness-of-fit chi-square test. The heterogeneity among study cohorts was evaluated using Review Manager 5 software (www.cc-ims.net/RevMan) and carried out with the Mantel-Haenszel method. A significant *I*^2^ statistic (*I*^2^ > 30%, *P* < 0.05) indicated heterogeneity for ORs across studies. The fixed-effects model was applied in current heterogeneity analyses.

The frequencies of alleles and genotypes were compared between cases and controls, and were assessed using Pearson chi-square test and logistic regression adjusting for age and sex, respectively. Odds ratios (ORs) with 95% confidence intervals (CIs) were calculated to estimate the relative risk for developing SLE or clinical/serologic manifestations. A stepwise (forward conditional) logistic regression analysis was performed to test the independence of the identified SNPs. LD and haplotype were calculated using online software SHEsis (http://analysis2.bio-x.cn/myAnalysis.php). The Mann Whitney test was applied for the analysis of serum C1q levels between two genotypic groups. Statistical analyses were conducted using SPSS 13.0 software (SPSS Inc., Chicago, IL). The false discovery rate (FDR, *q*-value) was applied for the multiple testing corrections. *P*-value ≤ 0.05 was considered nominally statistical significant. A *q*-value ≤ 0.10 was considered statistically significant.

## Electronic supplementary material

Supplementary Information
