# Supplementary material for: Investigation of C1-complex regions reveals new C1Q variants associated with protection from systemic lupus erythematosus, and affect its transcript abundance
Source: Sci Rep. 2018 May 23;8:8048. doi: 10.1038/s41598-018-26380-x (PMC5966390; doi:10.1038/s41598-018-26380-x)
Supplement: Supplementary file 1 — Supplementary Information [file 41598_2018_26380_MOESM1_ESM.pdf]

Investigation of C1-complex regions reveals new *C1Q* variants associated with protection from systemic lupus erythematosus, and affect its transcript abundance

Jianping Guo[1# *], Yanyan Gao[1, 2#], Yuxuan Wang[1], Yundong Zou[1], Yan Du[1], Cainan Luo[3], Yamei Shi[3], Yue Yang[1], Yue Yang[1], Xinyu Wu[1],Yin Su[1], Lijun Wu[3], Shi Chen[1*] and Zhanguo Li[1*]

[1] Department of Rheumatology and Immunology, Peking University People's Hospital, Beijing, China

[2] Department of Neurology，Children's Hospital Affiliated to Capital Institute of Pediatrics, Beijing, China

[3] Department of Rheumatology and Immunology, The People’s Hospital of Xinjiang Uygur Autonomous Region, Urumqi, China

[#] Jianping Guo and Yanyan Gao have equally contributed to this work.

[*] Jianping Guo, Shi Chen and Zhanguo Li are Co-corresponding authors.

Address correspondence to Jianping Guo MD PhD, Shi Chen, MD or Zhanguo Li, MD PhD, Department of Rheumatology and Immunology, Peking University People's Hospital, 11 South Xizhimen Street, Beijing 100044, China.

Tel: +86 (0)10 88324372

Fax: +86 (0)10 88324372

E-mail: jianping.guo@bjmu.edu.cn

SUPPLEMENTARY MATERIALS AND METHODS

Autoantibody detection

In two SLE cohorts, the autoantibodies antinuclear antibodies (ANA), anti-SSA /SSB antibodies, anti-double stranded DNA (dsDNA) antibodies, anti-Smith (Sm) antibodies, anti-cardiolipin antibodies (ACA), anti-RNP (ribonucleoprotein) antibodies, and anti-histone antibodies (AHA) were routinely measured. The data were available for all the in-patients and part of out-patients due to the incomplete records in electronic system in out-patient Dept.

ANA were detected by indirect immunofluorescence on HEp2 cells as substrate (Euroimmun, Lübeck, Germany) and considered positive at titre >1:40; Anti-dsDNA antibodies were measured with enzyme-linked immunosorbent assay (ELISA; Kexin Biotechnology Ltd. ShangHai, China). Values >100 IU/ml were assessed as positive. ACA and AHA were assessed with an immunoassay method by commercial microplate ELISA kits (Euroimmun). The precipitating antibodies to extractable nuclear antigens, including anti-SSA, anti-SSB, anti-Sm antibodies, and anti-RNP antibodies were determined by an immunoblot method (Euroimmun). Results were reported as positive or negative in relation to reference sera.

SUPPLEMENTARY RESULTS

Haplotype analysis shows consistent results supporting the protective effect of the four *C1Q* polymorphisms on SLE susceptibility

Similar results were also obtained from haplotype analysis. As shown in Supplementary Table S4, the haplotype constituted by the four 'protective' alleles'C-G-T-T' (rs680123–rs682658–rs653286–rs291985) displayed reduced risk contributed to SLE susceptibility (OR 0.84, *P* = 0.023). In two-maker haplotype analysis, the haplotype constituted by the two 'protective' alleles 'T-T' from rs653286–rs291985 also displayed reduced risk on SLE (OR 0.86, *P* = 0.036). In contrast, the haplotype 'C-G' (rs653286–rs291985) displayed an increased risk contributed to SLE susceptibility (OR 1.16, *P* = 0.036). As shown in Supplementary Figure S1, the variants rs680123 - rs682658, and rs653286 - rs291985 are almost in perfect LD (r^2 = 0.96/0.97 in controls/cases, respectively).

SUPPLEMENTARY FIGURES

Figure S1

A)

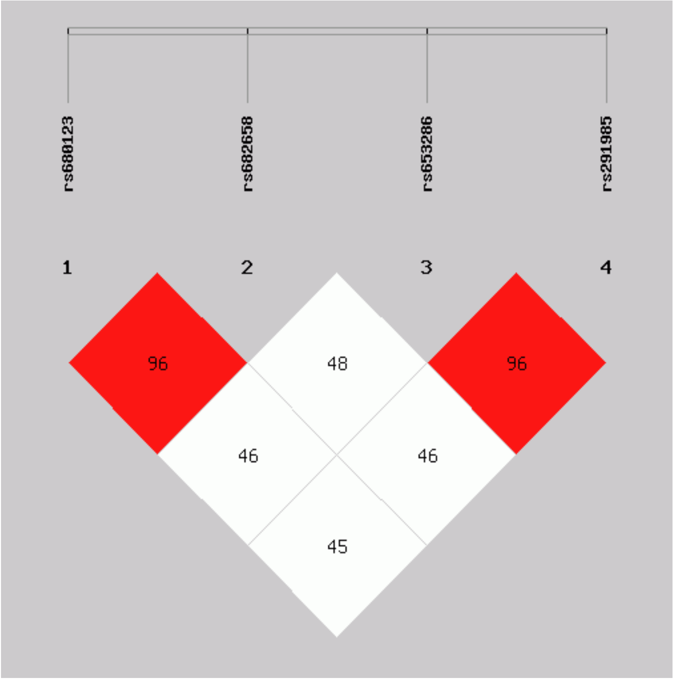

B)

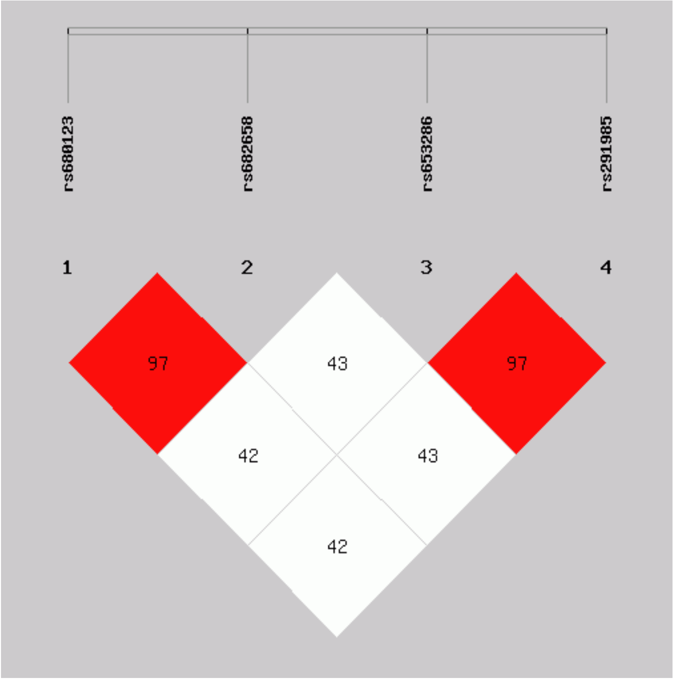

Figure S2

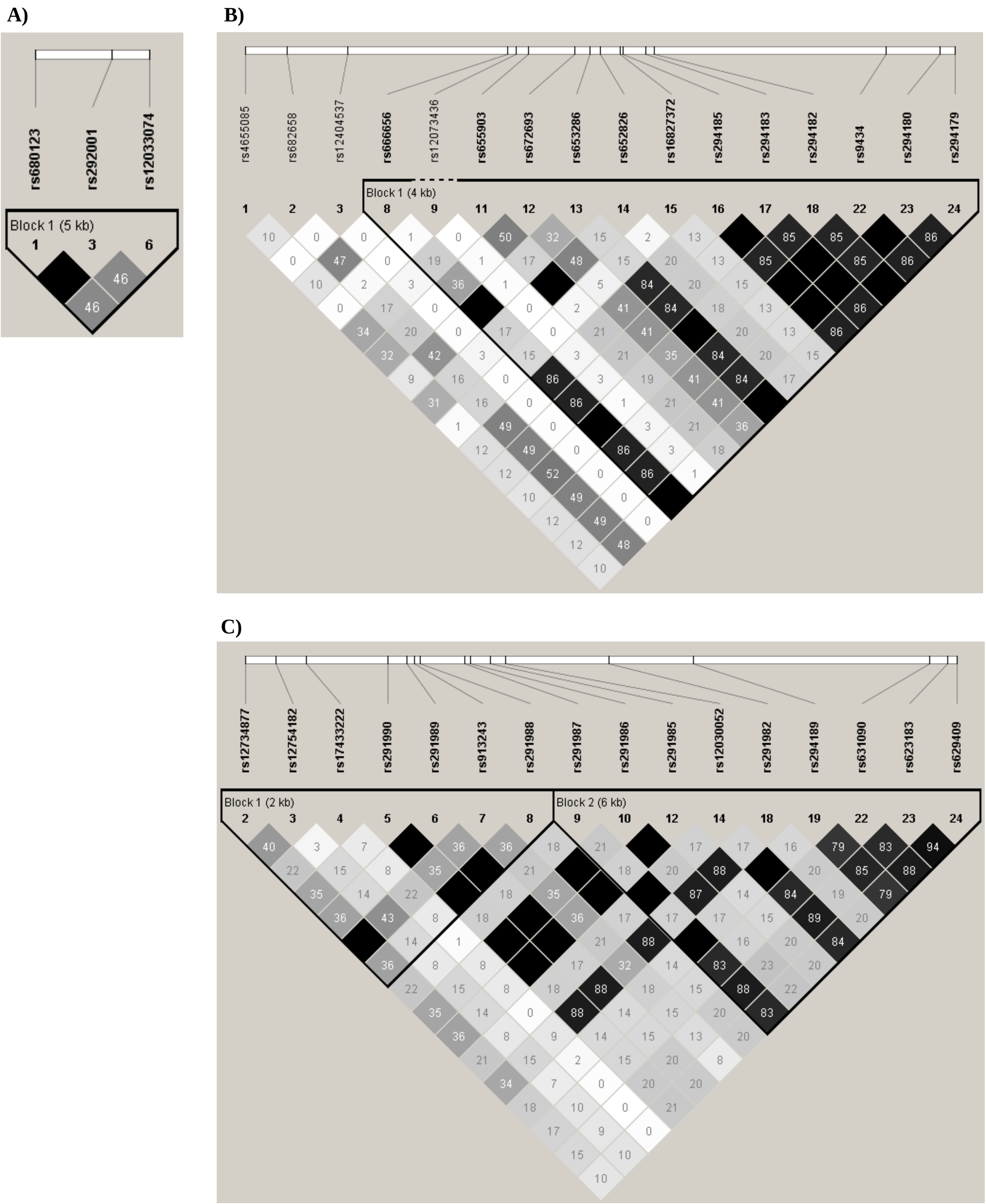
A)
rs680123
rs292001
rs12033074
Block 1 (5 kb)
1 3 6
B)
rs4655085
rs682658
rs12404537
rs666656
rs12073436
rs655903
rs672693
rs653286
rs652826
rs16827372
rs294185
rs294183
rs294182
rs9434
rs294180
rs294179
Block 1 (4 kb)
1 2 3 8 9 11 12 13 14 15 16 17 18 22 23 24
C)
rs12734877
rs12754182
rs17433222
rs291990
rs291989
rs913243
rs291988
rs291987
rs291986
rs291985
rs12030052
rs291982
rs294189
rs631090
rs623183
rs629409
Block 1 (2 kb)
Block 2 (6 kb)
2 3 4 5 6 7 8 9 10 12 14 18 19 22 23 24

D)

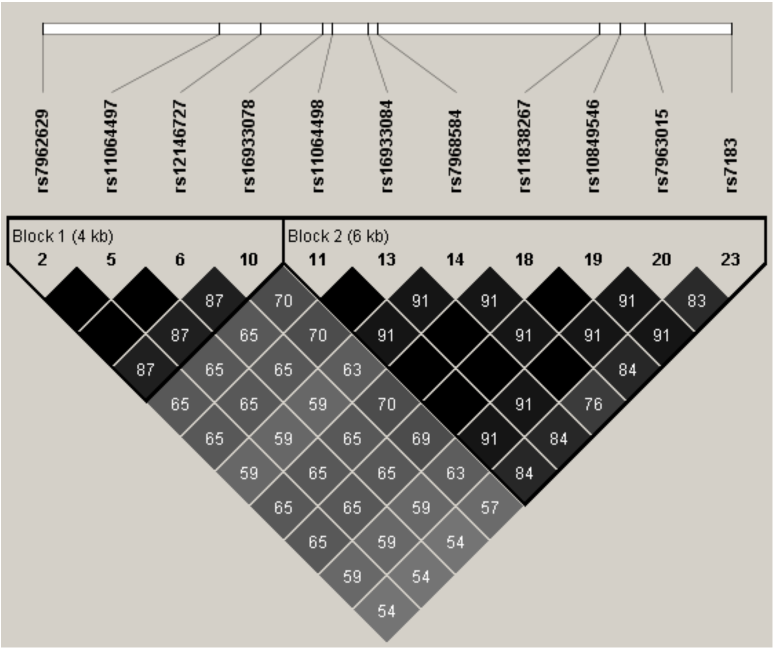
rs7962629
rs11064497
rs12146727
rs16933078
rs11064498
rs16933084
rs7968584
rs11838267
rs10849546
rs7963015
rs7183
Block 1 (4 kb)
Block 2 (6 kb)
2 5 6 10 11 13 14 18 19 20 23

E)

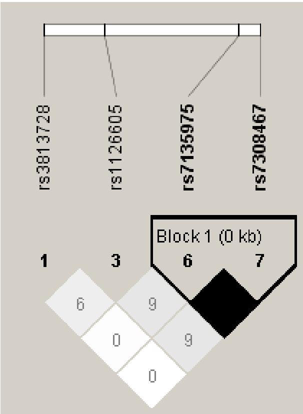
rs3813728
rs1126605
rs7135975
rs7308467
Block 1 (0 kb)
1 3 6 7

F)

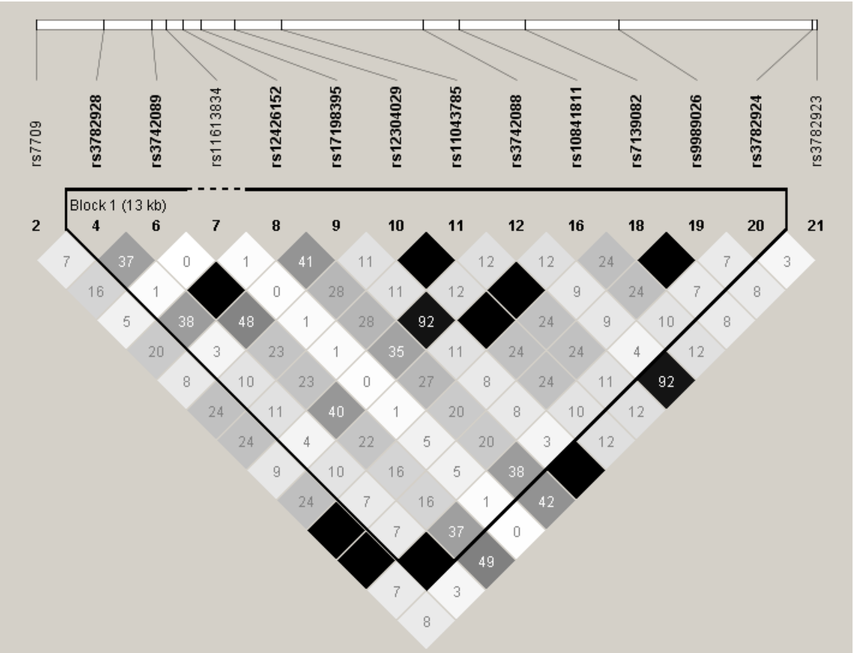
rs7709
rs3782928
rs3742089
rs11613834
rs12426152
rs17198395
rs12304029
rs11043785
rs3742088
rs10841811
rs7139082
rs9989026
rs3782924
rs3782923
Block 1 (13 kb)
2 4 6 7 8 9 10 11 12 16 18 19 20 21

Legends to Supplementary Figures

Figure S1 Linkage disequilibrium (LD) structure of SNPs rs680123, rs682658, rs653286, and rs291985 in *C1Q* genes in combined cohort. LD structure and haplotypes are determined using online software SHEsis (http://analysis2.bio-x.cn/myAnalysis.php). LD is measured using r^2 values and displayed in the squares. A) LD structure in healthy controls. B) LD structure in SLE cases.

Figure S2 Linkage disequilibrium (LD) structure for six C1-complex genes. LD structure and haplotypes are determined using using Haploview v4.2, according to HapMap phase III Chinese Han Beijing (CHB) panel (http://hapmap.ncbi.nlm.nih.gov/). LD is measured using r^2 values and displayed in the squares. A) LD structure of *C1QA*. B) LD structure of *C1QC*. C) LD structure of *C1QB*. D) LD structure of *C1S*. E) LD structure of *C1R*. F) LD structure of *C1RL*.

SUPPLEMENTARY TABLES

Table S1 Associations between additional 18 SNPs in C1 complex genes with SLE susceptibility in discovery cohort, logistic regression with adjustment for age and gender

Gene	SNP	Allele[a]	MAF[b] Cases/Cons	Allelic model OR(95% CI)	Allelic model *p*-value	Recessive model OR(95%CI)	Recessive model *p*-value	Dominant model OR(95% CI)	Dominant model *p*-value
C1QA	rs12033074	G/C	0.468/0.431	1.64 (0.95-1.43)	0.143	1.27 (0.87-1.84)	0.221	1.01(0.72-1.40)	0.970
C1QC	rs4655085	A/G	0.183/0.181	1.01(0.78-1.32)	0.922	1.01(0.46-2.24)	0.980	1.14(0.82-1.59)	0.434
C1QC	rs655903	G/A	0.278/0.260	1.10(0.87-1.39)	0.429	1.11(0.81-1.53)	0.511	1.09(0.79-1.49)	0.613
C1QC	rs672693	A/G	0.398/0.389	1.04(0.84-1.28)	0.732	1.09(0.71-1.68)	0.703	1.14(0.82-1.57)	0.439
C1QB	rs12754182	T/C	0.202/0.184	1.12(0.87-1.46)	0.375	0.76(0.27-2.09)	0.591	1.07(0.77-1.47)	0.702
C1QB	rs17433222	A/G	0.148/0.159	0.92(0.70-1.23)	0.578	1.18(0.68-2.06)	0.559	0.99(0.67-1.40)	0.947
C1QB	rs913243	T/G	0.441/0.416	1.11(0.90-1.36)	0.325	0.99(0.67-1.47)	0.965	1.16(0.83-1.61)	0.385
C1QB	rs629409	A/G	0.298/0.279	1.09(0.87-1.37)	0.425	1.35(0.78-2.32)	0.285	1.21(0.89-1.65)	0.234
C1S	rs7962629	G/A	0.096/0.117	0.80(0.58-1.11)	0.185	0.49(0.05-4.89)	0.547	1.39(0.89-2.20)	0.147
C1S	rs12146727	A/G	0.090/0.078	1.16(0.81-1.68)	0.418	0.49(0.05-4.90)	0.547	1.39(0.88-2.18)	0.158
C1S	rs7183	T/G	0.093/0.084	1.35(0.94-1.95)	0.104	0.75(0.11-4.96)	0.763	1.12(0.89-1.40)	0.346
C1R	rs3813728	T/C	0.148/0.124	1.23(0.91-1.65)	0.179	1.58(0.36-6.92)	0.544	1.21(0.84-1.73)	0.303
C1R	rs7135975	G/A	0.152/0.164	0.92(0.69-1.21)	0.545	1.16(0.43-3.13)	0.767	0.78(0.55-1.10)	0.149
C1RL	rs7709	C/A	0.238/0.250	0.94(0.74-1.19)	0.603	1.06(0.56-2.00)	0.863	0.94(0.69-1.29)	0.712
C1RL	rs3782928	A/G	0.250/0.161	0.92(0.69-1.22)	0.555	0.98(0.36-2.69)	0.965	0.78(0.55-1.11)	0.165
C1RL	rs3742089	G/A	0.295/0.295	1.00(0.80-1.25)	0.996	1.24(0.71-2.18)	0.448	0.97(0.71-1.33)	0.849
C1RL	rs12304029	C/G	0.451/0.434	1.07(0.87-1.31)	0.523	1.19(0.86-1.76)	0.400	0.99(0.71-1.39)	0.968
C1RL	rs3742088	G/T	0.160/0.151	1.07(0.81-1.42)	0.641	1.11(0.38-3.24)	0.848	1.23(0.87-1.73)	0.239

SLE: systemic lupus erythematosus; [a] Minor allele/Major allele, [b] Minor allele frequencies in discovery cohort (cases/controls); OR (95% CI): odds ratio (95% confidence interval).

Table S2 *P*-values and *q*-values of the 22 SNPs in association with SLE

Gene	SNP	Allele[a]	Allelic model		Recessive model		Dominant model	
			***p*-value**	***q*-value**	***p*-value**	***q*-value**	***p*-value**	***q*-value**
**Stage I**								
C1QA	rs680123	C/T	0.143	0.588	0.822	0.980	0.028	0.235
C1QC	rs682658	G/T	0.187	0.588	0.652	0.980	0.032	0.235
C1QC	rs653286	T/C	0.091	0.588	0.905	0.980	0.018	0.235
C1QB	rs291985	T/G	0.218	0.599	0.740	0.980	0.059	0.325
C1QA	rs12033074	G/C	0.143	0.588	0.221	0.980	0.970	0.970
C1QC	rs4655085	A/G	0.922	0.966	0.980	0.980	0.434	0.644
C1QC	rs655903	G/A	0.429	0.726	0.511	0.980	0.613	0.844
C1QC	rs672693	A/G	0.732	0.805	0.703	0.980	0.439	0.644
C1QB	rs12754182	T/C	0.375	0.726	0.591	0.980	0.702	0.870
C1QB	rs17433222	A/G	0.578	0.737	0.559	0.980	0.947	0.970
C1QB	rs913243	T/G	0.325	0.726	0.965	0.980	0.385	0.644
C1QB	rs629409	A/G	0.425	0.726	0.285	0.980	0.234	0.526
C1S	rs7962629	G/A	0.185	0.588	0.547	0.980	0.147	0.454
C1S	rs12146727	A/G	0.418	0.726	0.547	0.980	0.158	0.454
C1S	rs7183	T/G	0.104	0.588	0.763	0.980	0.346	0.634
C1R	rs3813728	T/C	0.179	0.588	0.544	0.980	0.303	0.606
C1R	rs7135975	G/A	0.545	0.737	0.767	0.980	0.149	0.454
C1RL	rs7709	C/A	0.603	0.737	0.863	0.980	0.712	0.870
C1RL	rs3782928	A/G	0.555	0.737	0.965	0.980	0.165	0.454
C1RL	rs3742089	G/A	0.996	0.996	0.448	0.980	0.849	0.970
C1RL	rs12304029	C/G	0.523	0.737	0.400	0.980	0.968	0.970
C1RL	rs3742088	G/T	0.641	0.742	0.848	0.980	0.239	0.526
**Stage II**								
C1QA	rs680123	C/T	0.394	0.489	0.833	0.960	0.186	0.240
C1QC	rs682658	G/T	0.489	0.489	0.772	0.960	0.240	0.240
C1QC	rs653286	T/C	0.138	0.276	0.960	0.960	0.045	0.090
C1QB	rs291985	T/G	0.098	0.276	0.940	0.960	0.019	0.076
**Combined**								
C1QA	rs680123	C/T	0.116	0.154	0.788	0.993	0.016	0.021
C1QC	rs682658	G/T	0.187	0.187	0.631	0.993	0.024	0.024
C1QC	rs653286	T/C	0.024	0.070	0.993	0.993	0.00245	0.00594
C1QB	rs291985	T/G	0.035	0.070	0.828	0.993	0.00297	0.00594

SLE: systemic lupus erythematosus; SNPs: single nucleotide polymorphisms.

[a] Minor allele frequencies in combined cohort (cases/controls).

Table S3 Haplotype analysis of four SNPs in SLE cases and healthy controls

Haplotype	SLE (%)	CON (%)	χ2	*P* value	OR (95%CI)
**1-2-3-4**					
C- G-T-T	26.1	29.4	5.184	0.023	0.84 (0.73-0.98)
T-T-C-G	57.8	55.1	2.606	0.106	1.12 (0.98-1.27)
C-G-C-G	8.2	7.1	1.287	0.257	1.15 (0.90-1.47)
T-T-T-T	6.6	6.9	0.091	0.763	0.96 (0.74-1.25)
**1-2**					
C-G	34.5	37.0	2.465	0.116	0.90 (0.78-1.03)
T-T	64.8	62.3	2.465	0.116	1.11 (0.97-1.28)
**3-4**					
T-T	33.1	36.4	4.392	0.036	0.86 (0.75-0.99)
C-G	66.2	62.8	4.392	0.036	1.16 (1.01-1.33)

SLE: systemic lupus erythematosus; OR (95% CI): odds ratio (95% confidence interval); variants order: rs680123 (1)–rs682658 (2)–rs653286 (3)–rs291985 (4).

All those frequencies less than 3% were ignored in analysis.

Table S4 Associations o SNP rs653286 polymorphisms with SLE clinical/serologic manifestations in case-only cohort, logistic regression adjusting for age and gender (dominant model)

Phenotype (n)*	Outcome[§]	Genotype frequency (%)		Patients only	
		TT+TC	CC	OR (95% CI)	*P* value
Rash (n=626)	+<br>–	211 (50.6)<br>117 (56.0)	206 (49.4)<br>92 (44.0)	0.80 (0.55-1.17)	0.25
Photosensitivity (n=576)	+<br>–	108 (50.7)<br>194 (52.9)	103 (49.3)<br>173 (47.1)	0.91 (0.63-1.32)	0.62
Arthritis (n=585)	+<br>–	218 (55.1)<br>93 (49.2)	178 (44.9)<br>96 (50.8)	1.33 (0.93-1.92)	0.12
Renal disorder (n= 420)	+<br>–	136 (51.3)<br>81 (52.3)	129 (48.7)<br>74 (47.7)	0.93 (0.63-1.38)	0.74
Neurological disorder (n=238)	+<br>–	11 (57.9)<br>131 (59.8)	8 (42.1)<br>88 (40.2)	0.91 (0.34-2.42)	0.85
Leukopenia (n= 403)	+<br>–	113 (50.7)<br>94 (52.2)	110 (49.3)<br>86 (47.8)	0.96 (0.64-1.45)	0.85
Complement depressed (n=427)	+<br>–	153 (49.4)<br>65 (55.6)	157 (50.6)<br>52 (44.4)	0.79 (0.50-1.23)	0.29
ANA (n=658)	+<br>–	324 (52.8)<br>22 (50.0)	290 (47.2)<br>22 (50.0)	1.20 (0.61-2.34)	0.60
Anti-dsDNA (n=600)	+<br>–	180 (48.3)<br>131 (57.7)	193 (51.7)<br>96 (42.3)	**0.68 (0.49-0.95)**	**0.024**
Anti-SSA (n=584)	+<br>–	140 (50.2)<br>167(54.8)	139 (49.8)<br>138 (45.2)	0.88 (0.63-1.22)	0.44
ACA (n=474)	+<br>–	47 (50.5)<br>199 (52.2)	46 (49.5)<br>182 (47.8)	0.91 (0.57-1.45)	0.70
AHA (n=373)	+<br>–	34 (47.2)<br>149 (49.5)	38 (52.8)<br>152 (50.5)	0.88 (0.53-1.49)	0.64

*: "n" indicating the number of cases in combined cohort when data were available; **§:** positivity of the phenotypes OR (95% CI): odds ratio (95% confidence interval). ANA: antinuclear autoantibodies; Anti-dsDNA: anti-double stranded DNA antibodies; Anti-SSA: anti-SSA antibody; ACA: anti-cardiolipin antibodies; AHA: anti-histone antibodies.

Table S5 Functional annotations of rs680123, rs682658, rs653286, and rs291985 by RegulomeDB

1. rs680123

A) Protein Binding					
**Method**	**Location**	**Bound Protein**	**Cell Type**	**Additional Info**	**Reference**
ChIP-seq	chr1:22961421..22962134	CREBBP	Jurkat		20019798

B) Chromatin structure				
**Method**	**Location**	**Cell Type**	**Additional Info**	**Reference**
DNase-seq	chr1:22961380..22961530	Jurkat		ENCODE
DNase-seq	chr1:22961386..22961736	Gm13976		ENCODE
DNase-seq	chr1:22961400..22961550	Hgf		ENCODE
DNase-seq	chr1:22961420..22961570	Cd20ro01778		ENCODE

C) Histone modifications					
**Method**	**Location**	**Chromatin State**	**Tissue Group**	**Tissue**	**Reference**
ChromHMM	chr1:22891000..23030200	Quiescent/Low	Blood & T-cell	Primary T helper naive cells from peripheral blood	REMC
ChromHMM	chr1:22906400..22979800	Quiescent/Low	Thymus	Fetal Thymus	REMC
ChromHMM	chr1:22907000..22965800	Repressed PolyComb	IMR90	IMR90 fetal lung fibroblasts Cell Line	REMC
ChromHMM	chr1:22914800..22977000	Weak Repressed PolyComb	Blood & T-cell	Primary T cells effector/memory enriched from peripheral blood	REMC
ChromHMM	chr1:22920200..23034000	Quiescent/Low	ES-deriv	H1 BMP4 Derived Mesendoderm Cultured Cells	REMC
ChromHMM	chr1:22921400..22967200	Weak Repressed PolyComb	Blood & T-cell	Primary T helper 17 cells PMA-I stimulated	REMC
ChromHMM	chr1:22924600..22964000	Repressed PolyComb	Mesench	Bone Marrow Derived Cultured Mesenchymal Stem Cells	REMC
ChromHMM	chr1:22926200..22976400	Weak Repressed PolyComb	Blood & T-cell	Primary T helper naive cells fromÃ‚ peripheralÃ‚ blood	REMC
ChromHMM	chr1:22926800..22968400	Weak Repressed PolyComb	HSC & B-cell	Primary B cells from peripheral blood	REMC
ChromHMM	chr1:22928600..22961800	Repressed PolyComb	HSC & B-cell	Primary hematopoietic stem cells G-CSF-mobilized Male	REMC
ChromHMM	chr1:22929000..22968400	Weak Repressed PolyComb	Blood & T-cell	Primary T regulatory cells fromÃ‚ peripheralÃ‚ blood	REMC
ChromHMM	chr1:22924400..22966000	Quiescent/Low	ENCODE	Dnd41 TCell Leukemia Cell Line	REMC
ChromHMM	chr1:22906200..22973000	Quiescent/Low	ENCODE	GM12878 Lymphoblastoid Cell Line	REMC
ChromHMM	chr1:22949200..23011000	Weak Repressed PolyComb	ENCODE	HMEC Mammary Epithelial Primary Cells	REMC
ChromHMM	chr1:22927600..22973800	Weak Repressed PolyComb	ENCODE	K562 Leukemia Cell Line	REMC
ChromHMM	chr1:22931400..22968400	Quiescent/Low	Blood & T-cell	Primary T helper memory cells from peripheral blood 2	REMC
ChromHMM	chr1:22938800..22970400	Weak Repressed PolyComb	Digestive	Esophagus	REMC

ChromHMM	chr1:22939200..22964800	Repressed PolyComb	Mesench	Mesenchymal Stem Cell Derived Chondrocyte Cultured Cells	REMC
ChromHMM	chr1:22940800..22962600	Weak Repressed PolyComb	Blood & T-cell	Primary T helper memory cells from peripheral blood 1	REMC
ChromHMM	chr1:22945600..22973400	Weak Repressed PolyComb	Blood & T-cell	Primary T CD8+ naive cells from peripheral blood	REMC
ChromHMM	chr1:22946000..22999400	Quiescent/Low	ES-deriv	H1 Derived Neuronal Progenitor Cultured Cells	REMC
ChromHMM	chr1:22950800..22962400	Repressed PolyComb	HSC & B-cell	Primary hematopoietic stem cells G-CSF-mobilized Female	REMC
ChromHMM	chr1:22951400..22964000	Repressed PolyComb	Myosat	Muscle Satellite Cultured Cells	REMC
ChromHMM	chr1:22952400..22967200	Weak Repressed PolyComb	Digestive	Sigmoid Colon	REMC
ChromHMM	chr1:22952400..22968000	Weak Repressed PolyComb	ENCODE	A549 EtOH 0.02pct Lung Carcinoma Cell Line	REMC
ChromHMM	chr1:22945200..22980800	Weak Repressed PolyComb	ENCODE	HUVEC Umbilical Vein Endothelial Primary Cells	REMC
ChromHMM	chr1:22952600..22963000	Weak Repressed PolyComb	Digestive	Duodenum Mucosa	REMC
ChromHMM	chr1:22952600..23020000	Weak Repressed PolyComb	Blood & T-cell	Primary T helper cells fromÃ,Â peripheralÃ,Â blood	REMC
ChromHMM	chr1:22953000..22963200	Weak Repressed PolyComb	Other	Fetal Kidney	REMC
ChromHMM	chr1:22953000..22968600	Weak Repressed PolyComb	Heart	Fetal Heart	REMC
ChromHMM	chr1:22953200..22963200	Weak Repressed PolyComb	Heart	Left Ventricle	REMC
ChromHMM	chr1:22953200..22963800	Repressed PolyComb	Neurosph	Cortex derived primary cultured neurospheres	REMC
ChromHMM	chr1:22953200..22976000	Weak Repressed PolyComb	HSC & B-cell	Primary neutrophils fromÃ,Â peripheralÃ,Â blood	REMC
ChromHMM	chr1:22953400..22963200	Weak Repressed PolyComb	Other	Liver	REMC
ChromHMM	chr1:22953400..22968000	Weak Repressed PolyComb	Brain	Brain Germinal Matrix	REMC
ChromHMM	chr1:22953400..22981200	Weak Repressed PolyComb	Sm. Muscle	Colon Smooth Muscle	REMC
ChromHMM	chr1:22953400..22983800	Quiescent/Low	Other	Pancreatic Islets	REMC
ChromHMM	chr1:22953600..22977200	Weak Repressed PolyComb	Blood & T-cell	Primary mononuclear cells fromÃ,Â peripheralÃ,Â blood	REMC
ChromHMM	chr1:22954000..22963200	Weak Repressed PolyComb	Brain	Brain Inferior Temporal Lobe	REMC
ChromHMM	chr1:22954200..22963200	Weak Repressed PolyComb	Brain	Brain Substantia Nigra	REMC
ChromHMM	chr1:22954200..22963200	Weak Repressed PolyComb	Digestive	Colonic Mucosa	REMC
ChromHMM	chr1:22954400..22963200	Weak Repressed PolyComb	Brain	Brain Anterior Caudate	REMC
ChromHMM	chr1:22954800..22963200	Weak Repressed PolyComb	Brain	Brain Hippocampus Middle	REMC
ChromHMM	chr1:22954800..22968400	Weak Repressed PolyComb	Brain	Brain Angular Gyrus	REMC
ChromHMM	chr1:22955000..22963400	Weak Repressed PolyComb	Digestive	Fetal Intestine Small	REMC
ChromHMM	chr1:22955000..23010000	Weak Repressed PolyComb	HSC & B-cell	Primary hematopoietic stem cells short term culture	REMC

ChromHMM	chr1:22955200..22968200	Weak Repressed PolyComb	Brain	Fetal Brain Male	REMC
ChromHMM	chr1:22955400..22963400	Weak Repressed PolyComb	Brain	Brain Cingulate Gyrus	REMC
ChromHMM	chr1:22953800..22968400	Weak Repressed PolyComb	Other	Placenta Amnion	REMC
ChromHMM	chr1:22953000..22963200	Weak Repressed PolyComb	Digestive	Rectal Mucosa Donor 29	REMC
ChromHMM	chr1:22953400..22963400	Weak Repressed PolyComb	Sm. Muscle	Rectal Smooth Muscle	REMC
ChromHMM	chr1:22954400..22974000	Weak Repressed PolyComb	Heart	Right Ventricle	REMC
ChromHMM	chr1:22954800..22999800	Weak Repressed PolyComb	Digestive	Stomach Mucosa	REMC
ChromHMM	chr1:22952800..22963400	Weak transcription	Other	Spleen	REMC
ChromHMM	chr1:22955400..22961800	Repressed PolyComb	ENCODE	HepG2 Hepatocellular Carcinoma Cell Line	REMC
ChromHMM	chr1:22952600..22980600	Weak Repressed PolyComb	ENCODE	NHDF-Ad Adult Dermal Fibroblast Primary Cells	REMC
ChromHMM	chr1:22955800..22961600	Quiescent/Low	Other	Pancreas	REMC
ChromHMM	chr1:22955800..22962000	Weak transcription	Digestive	Gastric	REMC
ChromHMM	chr1:22955800..22981800	Weak Repressed PolyComb	Epithelial	Foreskin Keratinocyte Primary Cells skin03	REMC
ChromHMM	chr1:22956200..22980000	Quiescent/Low	Other	Ovary	REMC
ChromHMM	chr1:22956800..22967400	Weak Repressed PolyComb	Blood & T-cell	Primary T helper cells PMA-I stimulated	REMC
ChromHMM	chr1:22957000..22961800	Weak Repressed PolyComb	Heart	Aorta	REMC
ChromHMM	chr1:22957000..22968400	Quiescent/Low	ES-deriv	hESC Derived CD184+ Endoderm Cultured Cells	REMC
ChromHMM	chr1:22958400..22976400	Weak Repressed PolyComb	Other	Fetal Lung	REMC
ChromHMM	chr1:22955800..22976400	Quiescent/Low	Muscle	Psoas Muscle	REMC
ChromHMM	chr1:22958400..22982400	Weak transcription	Heart	Right Atrium	REMC
ChromHMM	chr1:22956000..22993200	Quiescent/Low	Thymus	Thymus	REMC
ChromHMM	chr1:22956400..22964200	Repressed PolyComb	ENCODE	HeLa-S3 Cervical Carcinoma Cell Line	REMC
ChromHMM	chr1:22957000..22961800	Weak Repressed PolyComb	ENCODE	Monocytes-CD14+ RO01746 Primary Cells	REMC
ChromHMM	chr1:22960800..22994400	Weak Repressed PolyComb	ENCODE	NHEK-Epidermal Keratinocyte Primary Cells	REMC
ChromHMM	chr1:22960800..22977400	Weak Repressed PolyComb	ENCODE	NHLF Lung Fibroblast Primary Cells	REMC
ChromHMM	chr1:22961200..22975400	Weak Repressed PolyComb	ENCODE	Osteoblast Primary Cells	REMC
ChromHMM	chr1:22955800..23035800	Weak Repressed PolyComb	Digestive	Small Intestine	REMC
ChromHMM	chr1:22957200..22979800	Weak Repressed PolyComb	Epithelial	Foreskin Keratinocyte Primary Cells skin02	REMC
ChromHMM	chr1:22958600..22997800	Weak Repressed PolyComb	HSC & B-cell	Primary hematopoietic stem cells	REMC
ChromHMM	chr1:22958800..22968800	Weak Repressed PolyComb	Blood & T-cell	Primary T cells from cord blood	REMC
ChromHMM	chr1:22959800..22993200	Quiescent/Low	Blood & T-cell	Primary T CD8+ memory cells from peripheral blood	REMC
ChromHMM	chr1:22960200..22967400	Weak Repressed PolyComb	Epithelial	Breast Myoepithelial Primary Cells	REMC
ChromHMM	chr1:22961400..22994200	Quiescent/Low	HSC & B-cell	Primary B cells from cord blood	REMC
ChromHMM	chr1:22958000..22963000	Weak Repressed PolyComb	Digestive	Rectal Mucosa Donor 31	REMC

ChromHMM	chr1:22958000..22965600	Weak transcription	iPSC	iPS DF 6.9 Cell Line	REMC
ChromHMM	chr1:22958400..22963200	Weak Repressed PolyComb	Sm. Muscle	Duodenum Smooth Muscle	REMC
ChromHMM	chr1:22958600..22965600	Quiescent/Low	iPSC	iPS-20b Cell Line	REMC
ChromHMM	chr1:22958600..22966000	Quiescent/Low	ESC	ES-WA7 Cell Line	REMC
ChromHMM	chr1:22958800..22963400	Repressed PolyComb	Other	Fetal Adrenal Gland	REMC
ChromHMM	chr1:22958800..22965600	Quiescent/Low	iPSC	iPS-18 Cell Line	REMC
ChromHMM	chr1:22958800..22966200	Quiescent/Low	ESC	ES-I3 Cell Line	REMC
ChromHMM	chr1:22959200..22963200	Repressed PolyComb	Digestive	Fetal Stomach	REMC
ChromHMM	chr1:22959600..22962000	Enhancers	HSC & B-cell	Primary Natural Killer cells fromÃ,Â peripheralÃ,Â blood	REMC
ChromHMM	chr1:22959600..22963000	Repressed PolyComb	ENCODE	HSMM Skeletal Muscle Myoblasts Cell Line	REMC
ChromHMM	chr1:22959800..22961600	Repressed PolyComb	Muscle	Fetal Muscle Leg	REMC
ChromHMM	chr1:22959800..22961600	Repressed PolyComb	Other	Placenta	REMC
ChromHMM	chr1:22959800..22961800	Repressed PolyComb	ES-deriv	H1 Derived Mesenchymal Stem Cells	REMC
ChromHMM	chr1:22959800..22961800	Repressed PolyComb	Mesench	Mesenchymal Stem Cell Derived Adipocyte Cultured Cells	REMC
ChromHMM	chr1:22959800..22962000	Repressed PolyComb	Muscle	Skeletal Muscle Male	REMC
ChromHMM	chr1:22959800..22963200	Repressed PolyComb	Sm. Muscle	Stomach Smooth Muscle	REMC
ChromHMM	chr1:22959800..22966000	Repressed PolyComb	Epithelial	Foreskin Melanocyte Primary Cells skin03	REMC
ChromHMM	chr1:22960000..22964400	Repressed PolyComb	Epithelial	Breast variant Human Mammary Epithelial Cells (vHMEC)	REMC
ChromHMM	chr1:22960000..22965600	Repressed PolyComb	Epithelial	Foreskin Fibroblast Primary Cells skin01	REMC
ChromHMM	chr1:22960200..22964200	Repressed PolyComb	Epithelial	Foreskin Melanocyte Primary Cells skin01	REMC
ChromHMM	chr1:22960400..22962000	Heterochromatin	Other	Lung	REMC
ChromHMM	chr1:22960400..22962200	Heterochromatin	ESC	H1 Cell Line	REMC
ChromHMM	chr1:22960400..22962400	Repressed PolyComb	Neurosph	Ganglion Eminence derived primary cultured neurospheres	REMC
ChromHMM	chr1:22960400..22962600	Weak Repressed PolyComb	ENCODE	NH-A Astrocytes Primary Cells	REMC
ChromHMM	chr1:22960600..22961600	Repressed PolyComb	Muscle	Fetal Muscle Trunk	REMC
ChromHMM	chr1:22960800..22961800	Heterochromatin	ES-deriv	H9 Derived Neuronal Progenitor Cultured Cells	REMC
ChromHMM	chr1:22960800..22961800	Heterochromatin	Brain	Fetal Brain Female	REMC
ChromHMM	chr1:22960800..22962200	Heterochromatin	ESC	HUES6 Cell Line	REMC
ChromHMM	chr1:22961000..22961600	Repressed PolyComb	Brain	Brain Dorsolateral Prefrontal Cortex	REMC
ChromHMM	chr1:22961000..22961800	Heterochromatin	ESC	HUES48 Cell Line	REMC
ChromHMM	chr1:22961000..22961800	Heterochromatin	ESC	HUES64 Cell Line	REMC
ChromHMM	chr1:22961000..22961800	Heterochromatin	HSC & B-cell	Primary monocytes from peripheral blood	REMC
ChromHMM	chr1:22961000..22962000	Heterochromatin	ESC	H9 Cell Line	REMC

ChromHMM	chr1:22961000..22962000	Heterochromatin	ES-deriv	H9 Derived Neuron Cultured Cells	REMC
ChromHMM	chr1:22961000..22962000	ZNF genes & repeats	ESC	ES-UCSF4 Cell Line	REMC
ChromHMM	chr1:22961000..22963200	Heterochromatin	ES-deriv	H1 BMP4 Derived Trophoblast Cultured Cells	REMC
ChromHMM	chr1:22961200..22965800	Repressed PolyComb	Epithelial	Foreskin Fibroblast Primary Cells skin02	REMC
ChromHMM	chr1:22961400..22963600	Weak Repressed PolyComb	Digestive	Fetal Intestine Large	REMC
ChromHMM	chr1:22960800..22962000	Heterochromatin	ENCODE	HSMM cell derived Skeletal Muscle Myotubes Cell Line	REMC
ChromHMM	chr1:22961200..22961600	Bivalent Enhancer	Blood & T-cell	Primary T cells fromÃ,Â peripheralÃ,Â blood	REMC
ChromHMM	chr1:22961200..22962000	Heterochromatin	ES-deriv	hESC Derived CD56+ Ectoderm Cultured Cells	REMC
ChromHMM	chr1:22961200..22962200	Heterochromatin	iPSC	iPS-15b Cell Line	REMC
ChromHMM	chr1:22961200..22962400	Repressed PolyComb	Muscle	Skeletal Muscle Female	REMC
ChromHMM	chr1:22961200..22962600	Weak Repressed PolyComb	Mesench	Adipose Derived Mesenchymal Stem Cell Cultured Cells	REMC
ChromHMM	chr1:22961400..22962000	Heterochromatin	ES-deriv	hESC Derived CD56+ Mesoderm Cultured Cells	REMC
ChromHMM	chr1:22961400..22962000	Enhancers	Adipose	Adipose Nuclei	REMC
ChromHMM	chr1:22961400..22962400	ZNF genes & repeats	iPSC	iPS DF 19.11 Cell Line	REMC

B. rs682658

A) Protein Binding					
**Method**	**Location**	**Bound Protein**	**Cell Type**	**Additional Info**	**Reference**
ChIP-seq	chr1:22968433..22969029	ZNF263	HEK293-T-REx		ENCODE
ChIP-seq	chr1:22968414..22968878	MAZ	HeLa-S3		ENCODE
ChIP-seq	chr1:22968528..22968912	MYC	K562	ifng30	ENCODE
ChIP-seq	chr1:22968584..22968904	CTCF	WI-38		ENCODE
ChIP-seq	chr1:22968575..22968885	CTCF	HepG2		ENCODE
ChIP-seq	chr1:22968582..22968876	CTCF	Dnd41		ENCODE
ChIP-seq	chr1:22968579..22968862	CTCF	RPTEC		ENCODE
ChIP-seq	chr1:22968593..22968869	CTCF	HMEC		ENCODE
ChIP-seq	chr1:22968573..22968849	CTCF	MCF-7		ENCODE
ChIP-seq	chr1:22968601..22968864	CTCF	HeLa-S3		ENCODE
ChIP-seq	chr1:22968573..22968833	CTCF	HUVEC		ENCODE
ChIP-seq	chr1:22968574..22968833	CTCF	HCPEpiC		ENCODE

ChIP-seq	chr1:22968590..22968845	CTCF	HAc		ENCODE
ChIP-seq	chr1:22968579..22968832	CTCF	HBMEC		ENCODE
ChIP-seq	chr1:22968601..22968853	CTCF	SAEC		ENCODE
ChIP-seq	chr1:22968589..22968840	CTCF	AG09319		ENCODE
ChIP-seq	chr1:22968584..22968834	CTCF	NB4		ENCODE
ChIP-seq	chr1:22968595..22968843	CTCF	HSMMtube		ENCODE
ChIP-seq	chr1:22968593..22968840	CTCF	HFF-Myc		ENCODE
ChIP-seq	chr1:22968620..22968864	CTCF	HL-60		ENCODE
ChIP-seq	chr1:22968585..22968828	CTCF	NHEK		ENCODE
ChIP-seq	chr1:22968593..22968836	CTCF	HPAF		ENCODE
ChIP-seq	chr1:22968588..22968831	CTCF	HVMF		ENCODE
ChIP-seq	chr1:22968589..22968826	CTCF	GM12873		ENCODE
ChIP-seq	chr1:22968598..22968828	CTCF	NHDF-neo		ENCODE
ChIP-seq	chr1:22968595..22968823	CTCF	HA-sp		ENCODE
ChIP-seq	chr1:22968597..22968823	CTCF	HUVEC		ENCODE
ChIP-seq	chr1:22968603..22968823	CTCF	NHEK		ENCODE
ChIP-seq	chr1:22968602..22968818	CTCF	HFF		ENCODE
ChIP-seq	chr1:22968620..22968820	CTCF	MCF-7	estrogen	ENCODE
ChIP-seq	chr1:22968581..22968845	RAD21	GM12878		ENCODE
ChIP-seq	chr1:22968573..22968843	YY1	HCT-116		ENCODE
ChIP-seq	chr1:22968732..22969096	CTCF	HCM		ENCODE
ChIP-seq	chr1:22968701..22969125	CTCF	K562		ENCODE
ChIP-seq	chr1:22968716..22969032	MYC	NB4		ENCODE
ChIP-seq	chr1:22968629..22968815	CTCF	GM12872		ENCODE
ChIP-seq	chr1:22968614..22968815	CTCF	SK-N-SH		ENCODE
ChIP-seq	chr1:22968585..22968815	CTCF	HepG2		ENCODE
ChIP-seq	chr1:22968575..22968815	EGR1	GM12878		ENCODE

ChIP-seq	chr1:22968512..22968872	IRF1	K562	ifng6h	ENCODE
ChIP-seq	chr1:22968512..22968876	ELF1	GM12878		ENCODE
ChIP-seq	chr1:22968542..22968849	CTCF	K562		ENCODE
ChIP-seq	chr1:22968542..22968910	CTCF	Osteobl		ENCODE
ChIP-seq	chr1:22968548..22968852	FOXP2	PFSK-1		ENCODE
ChIP-seq	chr1:22968556..22968856	CTCF	HCM		ENCODE
ChIP-seq	chr1:22968566..22968838	CTCF	BJ		ENCODE
ChIP-seq	chr1:22968518..22968854	POLR2A	SK-N-MC		ENCODE
ChIP-seq	chr1:22968544..22968840	RCOR1	K562		ENCODE
ChIP-seq	chr1:22968529..22968853	REST	U87		ENCODE
ChIP-seq	chr1:22968557..22968827	SMC3	GM12878		ENCODE
ChIP-seq	chr1:22968556..22968826	SMC3	HepG2		ENCODE
ChIP-seq	chr1:22968565..22968885	SP1	GM12878		ENCODE
ChIP-seq	chr1:22968530..22968874	YY1	GM12878		ENCODE
ChIP-seq	chr1:22968535..22968865	YY1	K562		ENCODE
ChIP-seq	chr1:22968553..22968843	ZNF143	GM12878		ENCODE
ChIP-seq	chr1:22968559..22968849	ZNF143	K562		ENCODE

B) Chromatin structure

Method	Location	Cell Type	Additional Info	Reference
FAIRE	chr1:22968515..22968959	Helas3	Ifng4h	ENCODE
FAIRE	chr1:22968560..22968817	Gm12892		ENCODE
FAIRE	chr1:22968572..22968917	Huvec		ENCODE
FAIRE	chr1:22968580..22968836	Helas3	Ifna4h	ENCODE
DNase-seq	chr1:22967935..22969124	H7es		ENCODE
DNase-seq	chr1:22967958..22969212	Medullo		ENCODE
DNase-seq	chr1:22967958..22969212	Medullod341		ENCODE
DNase-seq	chr1:22968095..22968950	T47d		ENCODE
DNase-seq	chr1:22968095..22968950	T47d	Est10nm30m	ENCODE
DNase-seq	chr1:22968104..22969069	Sknsh		ENCODE
DNase-seq	chr1:22968158..22969138	K562		ENCODE
DNase-seq	chr1:22968172..22968930	Huh75		ENCODE
DNase-seq	chr1:22968172..22968935	Urotsa		ENCODE
DNase-seq	chr1:22968172..22968938	Huh7		ENCODE

DNase-seq	chr1:22968214..22969073	Hsmmfshd		ENCODE
DNase-seq	chr1:22968229..22968962	Osteobl		ENCODE
DNase-seq	chr1:22968214..22969634	Hsmm		ENCODE
DNase-seq	chr1:22968232..22969634	Hsmmemb		ENCODE
DNase-seq	chr1:22968264..22969959	Gm13976		ENCODE
DNase-seq	chr1:22968333..22969761	Gm19240		ENCODE
DNase-seq	chr1:22968239..22968951	K562	Sahactrl	ENCODE
DNase-seq	chr1:22968239..22969499	Gm12892		ENCODE
DNase-seq	chr1:22968241..22969579	Gm12891		ENCODE
DNase-seq	chr1:22968260..22968955	Hmec		ENCODE
DNase-seq	chr1:22968264..22969361	Cll		ENCODE
DNase-seq	chr1:22968266..22969076	Ips		ENCODE
DNase-seq	chr1:22968267..22968968	K562	Nabut	ENCODE
DNase-seq	chr1:22968267..22969122	Cd20ro01794		ENCODE
DNase-seq	chr1:22968278..22969164	Mcf7	Hypoxlaccon	ENCODE
DNase-seq	chr1:22968278..22969164	Mcf7	Hypoxlac	ENCODE
DNase-seq	chr1:22968278..22969164	Mcf7		ENCODE
DNase-seq	chr1:22968281..22968911	Gliobla		ENCODE
DNase-seq	chr1:22968286..22968985	Helas3		ENCODE
DNase-seq	chr1:22968288..22968923	Monocd14		ENCODE
DNase-seq	chr1:22968289..22968925	Lncap		ENCODE
DNase-seq	chr1:22968291..22968930	Frontalcortexoc		ENCODE
DNase-seq	chr1:22968307..22969085	Gm19239		ENCODE
DNase-seq	chr1:22968313..22968975	H1hesc		ENCODE
DNase-seq	chr1:22968316..22969001	Hek293t		ENCODE
DNase-seq	chr1:22968316..22969491	Chorion		ENCODE
DNase-seq	chr1:22968319..22968985	Gm12878		ENCODE
DNase-seq	chr1:22968323..22968950	Heartoc		ENCODE
DNase-seq	chr1:22968324..22969008	Ipscwru1		ENCODE
DNase-seq	chr1:22968325..22968925	Lncap	Andro	ENCODE
DNase-seq	chr1:22968325..22968947	K562	Saha1u72hr	ENCODE
DNase-seq	chr1:22968330..22968904	UrotsaUt189		ENCODE
DNase-seq	chr1:22968335..22969069	Imr90		ENCODE
DNase-seq	chr1:22968335..22969085	Gm13977		ENCODE
DNase-seq	chr1:22968337..22968959	Fibrobl		ENCODE
DNase-seq	chr1:22968337..22968959	Fibroblgm03348		ENCODE
DNase-seq	chr1:22968337..22968959	Fibroblgm03348	Lentimyod	ENCODE
DNase-seq	chr1:22968345..22969197	Gm19238		ENCODE
DNase-seq	chr1:22968349..22968934	Mcf7	Randshrna	ENCODE
DNase-seq	chr1:22968350..22968953	Hepg2		ENCODE
DNase-seq	chr1:22968358..22968952	Ecc1	Est10nm30m	ENCODE
DNase-seq	chr1:22968358..22969108	Hsmmt		ENCODE
DNase-seq	chr1:22968359..22968885	8988t		ENCODE
DNase-seq	chr1:22968362..22969011	Cerebrumfrontaloc		ENCODE
DNase-seq	chr1:22968365..22968912	Ishikawa	Tam10030	ENCODE
DNase-seq	chr1:22968365..22968917	Nhek		ENCODE
DNase-seq	chr1:22968366..22969019	Gm10248		ENCODE
DNase-seq	chr1:22968371..22968942	Mel2183		ENCODE
DNase-seq	chr1:22968374..22968945	Fibroblgm03348	Lenticon	ENCODE
DNase-seq	chr1:22968376..22968945	K562G2mphase		ENCODE
DNase-seq	chr1:22968377..22968977	Ipsnihi11		ENCODE
DNase-seq	chr1:22968379..22968953	Ipsnihi7		ENCODE
DNase-seq	chr1:22968381..22968892	Ishikawa	Est10nm30m	ENCODE
DNase-seq	chr1:22968387..22968931	Stellate		ENCODE
DNase-seq	chr1:22968388..22968916	Gcbcell		ENCODE
DNase-seq	chr1:22968388..22968972	Adultcd4th1		ENCODE

DNase-seq	chr1:22968389..22968947	K562G1phase		ENCODE
DNase-seq	chr1:22968391..22968949	Helas3	Ifna4h	ENCODE
DNase-seq	chr1:22968391..22968950	Cerebellumoc		ENCODE
DNase-seq	chr1:22968395..22969150	Panislets		ENCODE
DNase-seq	chr1:22968397..22968916	Melano		ENCODE
DNase-seq	chr1:22968397..22968916	Olfneurosphere		ENCODE
DNase-seq	chr1:22968403..22968911	Rwpe1		ENCODE
DNase-seq	chr1:22968404..22968898	A549		ENCODE
DNase-seq	chr1:22968407..22969002	Naivebcell		ENCODE
DNase-seq	chr1:22968408..22968970	Phte		ENCODE
DNase-seq	chr1:22968412..22968977	Huvec		ENCODE
DNase-seq	chr1:22968409..22968932	Fibropag20443		ENCODE
DNase-seq	chr1:22968409..22968932	Fibrop		ENCODE
DNase-seq	chr1:22968410..22968915	H9es		ENCODE
DNase-seq	chr1:22968411..22968924	Panisd		ENCODE
DNase-seq	chr1:22968413..22968921	Ecc1	Dm002p1h	ENCODE
DNase-seq	chr1:22968424..22968925	Fibropag08396		ENCODE
DNase-seq	chr1:22968425..22968938	Gm20000		ENCODE
DNase-seq	chr1:22968431..22968888	Htr8		ENCODE
DNase-seq	chr1:22968431..22968899	Gm18507		ENCODE
DNase-seq	chr1:22968435..22968844	Hepatocytes		ENCODE
DNase-seq	chr1:22968440..22968932	Adultcd4th0		ENCODE
DNase-seq	chr1:22968441..22968874	Mcf7	Ctcfshrna	ENCODE
DNase-seq	chr1:22968444..22968896	Fibropag08395		ENCODE
DNase-seq	chr1:22968447..22968936	Gm10266		ENCODE
DNase-seq	chr1:22968449..22968901	Progfib		ENCODE
DNase-seq	chr1:22968457..22968909	Psoasmuscleoc		ENCODE
DNase-seq	chr1:22968461..22968905	Myometr		ENCODE
DNase-seq	chr1:22968470..22968882	Hpde6e6e7		ENCODE
DNase-seq	chr1:22968507..22968852	Aosmc	Serumfree	ENCODE

C) Histone modifications					
Method	Location	Chromatin State	Tissue Group	Tissue	Reference
ChromHMM	chr1:22891000..23030200	Quiescent/Low	Blood & T-cell	Primary T helper naive cells from peripheral blood	REMC
ChromHMM	chr1:22906400..22979800	Quiescent/Low	Thymus	Fetal Thymus	REMC
ChromHMM	chr1:22914800..22977000	Weak Repressed PolyComb	Blood & T-cell	Primary T cells effector/memory enriched from peripheral blood	REMC
ChromHMM	chr1:22920200..23034000	Quiescent/Low	ES-deriv	H1 BMP4 Derived Mesendoderm Cultured Cells	REMC
ChromHMM	chr1:22926200..22976400	Weak Repressed PolyComb	Blood & T-cell	Primary T helper naive cells fromÃ,Â peripheralÃ,Â blood	REMC
ChromHMM	chr1:22906200..22973000	Quiescent/Low	ENCODE	GM12878 Lymphoblastoid Cell Line	REMC
ChromHMM	chr1:22949200..23011000	Weak Repressed PolyComb	ENCODE	HMEC Mammary Epithelial Primary Cells	REMC
ChromHMM	chr1:22927600..22973800	Weak Repressed PolyComb	ENCODE	K562 Leukemia Cell Line	REMC
ChromHMM	chr1:22938800..22970400	Weak Repressed PolyComb	Digestive	Esophagus	REMC
ChromHMM	chr1:22945600..22973400	Weak Repressed PolyComb	Blood & T-cell	Primary T CD8+ naive cells from peripheral blood	REMC
ChromHMM	chr1:22946000..22999400	Quiescent/Low	ES-deriv	H1 Derived Neuronal Progenitor Cultured Cells	REMC
ChromHMM	chr1:22945200..22980800	Weak Repressed PolyComb	ENCODE	HUVEC Umbilical Vein Endothelial Primary Cells	REMC
ChromHMM	chr1:22952600..23020000	Weak Repressed PolyComb	Blood & T-cell	Primary T helper cells fromÃ,Â peripheralÃ,Â blood	REMC
ChromHMM	chr1:22953200..22976000	Weak Repressed PolyComb	HSC & B-cell	Primary neutrophils fromÃ,Â peripheralÃ,Â blood	REMC
ChromHMM	chr1:22953400..22981200	Weak Repressed PolyComb	Sm. Muscle	Colon Smooth Muscle	REMC
ChromHMM	chr1:22953400..22983800	Quiescent/Low	Other	Pancreatic Islets	REMC
ChromHMM	chr1:22953600..22977200	Weak Repressed PolyComb	Blood & T-cell	Primary mononuclear cells fromÃ,Â peripheralÃ,Â blood	REMC
ChromHMM	chr1:22955000..23010000	Weak Repressed PolyComb	HSC & B-cell	Primary hematopoietic stem cells short term culture	REMC
ChromHMM	chr1:22954400..22974000	Weak Repressed PolyComb	Heart	Right Ventricle	REMC
ChromHMM	chr1:22954800..22999800	Weak Repressed PolyComb	Digestive	Stomach Mucosa	REMC
ChromHMM	chr1:22952600..22980600	Weak Repressed PolyComb	ENCODE	NHDF-Ad Adult Dermal Fibroblast Primary Cells	REMC
ChromHMM	chr1:22955800..22981800	Weak Repressed PolyComb	Epithelial	Foreskin Keratinocyte Primary Cells skin03	REMC
ChromHMM	chr1:22956200..22980000	Quiescent/Low	Other	Ovary	REMC
ChromHMM	chr1:22958400..22976400	Weak Repressed PolyComb	Other	Fetal Lung	REMC
ChromHMM	chr1:22961800..22973000	Quiescent/Low	Heart	Aorta	REMC
ChromHMM	chr1:22962000..22971600	Quiescent/Low	Digestive	Gastric	REMC
ChromHMM	chr1:22955800..22976400	Quiescent/Low	Muscle	Psoas Muscle	REMC
ChromHMM	chr1:22958400..22982400	Weak transcription	Heart	Right Atrium	REMC
ChromHMM	chr1:22956000..22993200	Quiescent/Low	Thymus	Thymus	REMC
ChromHMM	chr1:22964200..22973800	Weak Repressed PolyComb	ENCODE	HeLa-S3 Cervical Carcinoma Cell Line	REMC

ChromHMM	chr1:22963400..22979000	Weak Repressed PolyComb	ENCODE	HSMM Skeletal Muscle Myoblasts Cell Line	REMC
ChromHMM	chr1:22960800..22994400	Weak Repressed PolyComb	ENCODE	NHEK-Epidermal Keratinocyte Primary Cells	REMC
ChromHMM	chr1:22960800..22977400	Weak Repressed PolyComb	ENCODE	NHLF Lung Fibroblast Primary Cells	REMC
ChromHMM	chr1:22961200..22975400	Weak Repressed PolyComb	ENCODE	Osteoblast Primary Cells	REMC
ChromHMM	chr1:22955800..23035800	Weak Repressed PolyComb	Digestive	Small Intestine	REMC
ChromHMM	chr1:22957200..22979800	Weak Repressed PolyComb	Epithelial	Foreskin Keratinocyte Primary Cells skin02	REMC
ChromHMM	chr1:22958600..22997800	Weak Repressed PolyComb	HSC & B-cell	Primary hematopoietic stem cells	REMC
ChromHMM	chr1:22959800..22993200	Quiescent/Low	Blood & T-cell	Primary T CD8+ memory cells from peripheral blood	REMC
ChromHMM	chr1:22961400..22994200	Quiescent/Low	HSC & B-cell	Primary B cells from cord blood	REMC
ChromHMM	chr1:22962000..22982000	Quiescent/Low	ES-deriv	hESC Derived CD56+ Ectoderm Cultured Cells	REMC
ChromHMM	chr1:22962000..22997000	Quiescent/Low	ESC	H9 Cell Line	REMC
ChromHMM	chr1:22962400..22982000	Weak Repressed PolyComb	Neurosph	Ganglion Eminence derived primary cultured neurospheres	REMC
ChromHMM	chr1:22962600..22995600	Quiescent/Low	Blood & T-cell	Primary T helper memory cells from peripheral blood 1	REMC
ChromHMM	chr1:22966000..23067800	Weak Repressed PolyComb	ENCODE	Dnd41 TCell Leukemia Cell Line	REMC
ChromHMM	chr1:22964000..22970400	Weak Repressed PolyComb	Digestive	Rectal Mucosa Donor 31	REMC
ChromHMM	chr1:22964000..22971600	Weak Repressed PolyComb	Myosat	Muscle Satellite Cultured Cells	REMC
ChromHMM	chr1:22964000..22973200	Weak Repressed PolyComb	Mesench	Bone Marrow Derived Cultured Mesenchymal Stem Cells	REMC
ChromHMM	chr1:22964200..22971200	Weak Repressed PolyComb	Other	Liver	REMC
ChromHMM	chr1:22964400..22970200	Weak transcription	Heart	Left Ventricle	REMC
ChromHMM	chr1:22965000..22971400	Weak Repressed PolyComb	Sm. Muscle	Rectal Smooth Muscle	REMC
ChromHMM	chr1:22968200..22969800	Bivalent Enhancer	Muscle	Skeletal Muscle Male	REMC
ChromHMM	chr1:22963400..22972800	Weak Repressed PolyComb	ENCODE	HSMM cell derived Skeletal Muscle Myotubes Cell Line	REMC
ChromHMM	chr1:22965000..22995800	Quiescent/Low	HSC & B-cell	Primary monocytes from peripheral blood	REMC
ChromHMM	chr1:22965400..22969600	Weak Repressed PolyComb	ES-deriv	H9 Derived Neuron Cultured Cells	REMC
ChromHMM	chr1:22965600..22976200	Weak Repressed PolyComb	Other	Fetal Kidney	REMC
ChromHMM	chr1:22965800..22970200	Weak Repressed PolyComb	Digestive	Rectal Mucosa Donor 29	REMC
ChromHMM	chr1:22966000..22974000	Weak transcription	Other	Lung	REMC
ChromHMM	chr1:22966000..22999800	Quiescent/Low	ESC	HUES48 Cell Line	REMC

ChromHMM	chr1:22966200..22970000	Weak Repressed PolyComb	iPSC	iPS-15b Cell Line	REMC
ChromHMM	chr1:22966200..22992400	Weak Repressed PolyComb	ESC	ES-WA7 Cell Line	REMC
ChromHMM	chr1:22966200..22999600	Quiescent/Low	iPSC	iPS-18 Cell Line	REMC
ChromHMM	chr1:22966600..22978600	Quiescent/Low	ES-deriv	hESC Derived CD56+ Mesoderm Cultured Cells	REMC
ChromHMM	chr1:22967200..22969200	Bivalent Enhancer	Muscle	Fetal Muscle Trunk	REMC
ChromHMM	chr1:22967400..22969200	Enhancers	iPSC	iPS DF 19.11 Cell Line	REMC
ChromHMM	chr1:22967800..22969200	Weak Repressed PolyComb	Epithelial	Foreskin Melanocyte Primary Cells skin03	REMC
ChromHMM	chr1:22967800..22969800	Bivalent Enhancer	Digestive	Duodenum Mucosa	REMC
ChromHMM	chr1:22968000..22969400	Bivalent Enhancer	Other	Placenta	REMC
ChromHMM	chr1:22968000..22969800	Enhancers	Adipose	Adipose Nuclei	REMC
ChromHMM	chr1:22968400..22992400	Quiescent/Low	iPSC	iPS DF 6.9 Cell Line	REMC
ChromHMM	chr1:22968400..22999600	Quiescent/Low	iPSC	iPS-20b Cell Line	REMC
ChromHMM	chr1:22968800..23003200	Weak Repressed PolyComb	Digestive	Sigmoid Colon	REMC
ChromHMM	chr1:22968000..22973400	Weak transcription	Other	Spleen	REMC
ChromHMM	chr1:22966000..22979000	Quiescent/Low	ENCODE	Monocytes-CD14+ RO01746 Primary Cells	REMC
ChromHMM	chr1:22965400..22979200	Weak Repressed PolyComb	ENCODE	NH-A Astrocytes Primary Cells	REMC
ChromHMM	chr1:22968200..22969000	Bivalent Enhancer	Epithelial	Breast Myoepithelial Primary Cells	REMC
ChromHMM	chr1:22968200..22969000	Bivalent Enhancer	Epithelial	Foreskin Fibroblast Primary Cells skin01	REMC
ChromHMM	chr1:22968400..22969200	Bivalent Enhancer	Epithelial	Foreskin Fibroblast Primary Cells skin02	REMC
ChromHMM	chr1:22968200..22969000	Enhancers	Brain	Brain Anterior Caudate	REMC
ChromHMM	chr1:22968200..22969000	Bivalent Enhancer	Brain	Brain Dorsolateral Prefrontal Cortex	REMC
ChromHMM	chr1:22968400..22969200	Enhancers	Brain	Brain Substantia Nigra	REMC
ChromHMM	chr1:22968200..22969200	Enhancers	Muscle	Fetal Muscle Leg	REMC
ChromHMM	chr1:22968200..22969000	Enhancers	Other	Pancreas	REMC
ChromHMM	chr1:22968200..22969000	Bivalent/Poised TSS	Sm. Muscle	Stomach Smooth Muscle	REMC
ChromHMM	chr1:22968600..22969600	Repressed PolyComb	Mesench	Adipose Derived Mesenchymal Stem Cell Cultured Cells	REMC
ChromHMM	chr1:22968600..22969800	Repressed PolyComb	Epithelial	Breast variant Human Mammary Epithelial Cells (vHMEC)	REMC
ChromHMM	chr1:22968600..22969800	Repressed PolyComb	HSC & B-cell	Primary hematopoietic stem cells G-CSF-mobilized Male	REMC
ChromHMM	chr1:22968600..22970000	Repressed PolyComb	ES-deriv	H9 Derived Neuronal Progenitor Cultured Cells	REMC
ChromHMM	chr1:22968600..22970200	Repressed PolyComb	Blood & T-cell	Primary T helper 17 cells PMA-I stimulated	REMC
ChromHMM	chr1:22968600..22970800	Weak Repressed PolyComb	ENCODE	HepG2 Hepatocellular Carcinoma Cell Line	REMC
ChromHMM	chr1:22968800..22969800	Repressed PolyComb	Blood & T-cell	Primary T cells from cord blood	REMC
ChromHMM	chr1:22968800..22970200	Repressed PolyComb	Other	Fetal Adrenal Gland	REMC
ChromHMM	chr1:22968800..22970400	Bivalent Enhancer	Epithelial	Foreskin Melanocyte Primary Cells skin01	REMC

ChromHMM	chr1:22968400..22969000	Bivalent Enhancer	ES-deriv	H1 Derived Mesenchymal Stem Cells	REMC
ChromHMM	chr1:22968400..22969000	Enhancers	Blood & T-cell	Primary T helper memory cells from peripheral blood 2	REMC
ChromHMM	chr1:22968400..22969000	Enhancers	HSC & B-cell	Primary Natural Killer cells fromÃ,Â peripheralÃ,Â blood	REMC
ChromHMM	chr1:22968400..22969000	Bivalent Enhancer	Neurosph	Cortex derived primary cultured neurospheres	REMC
ChromHMM	chr1:22968400..22969000	Bivalent Enhancer	Brain	Brain Angular Gyrus	REMC
ChromHMM	chr1:22968600..22969000	Bivalent Enhancer	Brain	Brain Cingulate Gyrus	REMC
ChromHMM	chr1:22968600..22969000	Repressed PolyComb	Sm. Muscle	Duodenum Smooth Muscle	REMC
ChromHMM	chr1:22968400..22969000	Bivalent Enhancer	Digestive	Fetal Intestine Large	REMC
ChromHMM	chr1:22968400..22969000	Enhancers	Digestive	Fetal Intestine Small	REMC
ChromHMM	chr1:22968800..22969000	Flanking Bivalent TSS/Enh	Muscle	Skeletal Muscle Female	REMC
ChromHMM	chr1:22968600..22969000	Repressed PolyComb	Blood & T-cell	Primary T helper cells PMA-I stimulated	REMC
ChromHMM	chr1:22968600..22969200	Repressed PolyComb	Mesench	Mesenchymal Stem Cell Derived Adipocyte Cultured Cells	REMC
ChromHMM	chr1:22968800..22969000	Repressed PolyComb	Blood & T-cell	Primary T regulatory cells fromÃ,Â peripheralÃ,Â blood	REMC
ChromHMM	chr1:22968800..22969400	Repressed PolyComb	Digestive	Colonic Mucosa	REMC
ChromHMM	chr1:22968600..22971600	Weak Repressed PolyComb	Brain	Brain Inferior Temporal Lobe	REMC
ChromHMM	chr1:22968600..22971600	Repressed PolyComb	Digestive	Fetal Stomach	REMC
ChromHMM	chr1:22968600..22972200	Weak transcription	ES-deriv	H1 BMP4 Derived Trophoblast Cultured Cells	REMC
ChromHMM	chr1:22968600..22972600	Quiescent/Low	ESC	H1 Cell Line	REMC
ChromHMM	chr1:22968600..22972600	Repressed PolyComb	IMR90	IMR90 fetal lung fibroblasts Cell Line	REMC
ChromHMM	chr1:22968600..22972600	Active TSS	Brain	Brain Hippocampus Middle	REMC
ChromHMM	chr1:22968600..22975200	Quiescent/Low	ESC	ES-UCSF4 Cell Line	REMC
ChromHMM	chr1:22968600..22976200	Weak Repressed PolyComb	Brain	Fetal Brain Male	REMC
ChromHMM	chr1:22968600..22978200	Weak Repressed PolyComb	Mesench	Mesenchymal Stem Cell Derived Chondrocyte Cultured Cells	REMC
ChromHMM	chr1:22968600..22978400	Quiescent/Low	Heart	Fetal Heart	REMC
ChromHMM	chr1:22968600..22979200	Quiescent/Low	Brain	Brain Germinal Matrix	REMC
ChromHMM	chr1:22968600..22979600	Quiescent/Low	ENCODE	A549 EtOH 0.02pct Lung Carcinoma Cell Line	REMC
ChromHMM	chr1:22968600..22979800	Quiescent/Low	Brain	Fetal Brain Female	REMC
ChromHMM	chr1:22968600..22981000	Weak Repressed PolyComb	HSC & B-cell	Primary hematopoietic stem cells G-CSF-mobilized Female	REMC
ChromHMM	chr1:22968600..22985000	Weak Repressed PolyComb	ESC	HUES6 Cell Line	REMC
ChromHMM	chr1:22968600..22985600	Weak Repressed PolyComb	ES-deriv	hESC Derived CD184+ Endoderm Cultured Cells	REMC
ChromHMM	chr1:22968600..22992800	Weak Repressed PolyComb	ESC	ES-I3 Cell Line	REMC
ChromHMM	chr1:22968600..22999800	Quiescent/Low	ESC	HUES64 Cell Line	REMC
ChromHMM	chr1:22968800..22972600	Repressed PolyComb	Blood & T-cell	Primary T cells fromÃ,Â peripheralÃ,Â blood	REMC
ChromHMM	chr1:22968800..22973200	Weak Repressed PolyComb	HSC & B-cell	Primary B cells from peripheral blood	REMC

ChromHMM	chr1:22968800..22978000	Quiescent/Low	Other	Placenta Amnion	REMC

C. rs653286

A) Chromatin structure				
**Method**	**Location**	**Cell Type**	**Additional Info**	**Reference**
DNase-seq	chr1:22971141..22971758	Chorion		ENCODE
DNase-seq	chr1:22971498..22971811	Gm13976		ENCODE
DNase-seq	chr1:22971518..22971823	Gm12892		ENCODE
DNase-seq	chr1:22971557..22971736	Monocd14		ENCODE
DNase-seq	chr1:22971565..22971818	Huh75		ENCODE
DNase-seq	chr1:22971565..22971818	Huh7		ENCODE
DNase-seq	chr1:22971566..22971714	Hepatocytes		ENCODE

B) Histone modifications					
**Method**	**Location**	**Chromatin State**	**Tissue Group**	**Tissue**	**Reference**
ChromHMM	chr1:22891000..23030200	Quiescent/Low	Blood & T-cell	Primary T helper naive cells from peripheral blood	REMC
ChromHMM	chr1:22906400..22979800	Quiescent/Low	Thymus	Fetal Thymus	REMC
ChromHMM	chr1:22914800..22977000	Weak Repressed PolyComb	Blood & T-cell	Primary T cells effector/memory enriched from peripheral blood	REMC
ChromHMM	chr1:22920200..23034000	Quiescent/Low	ES-deriv	H1 BMP4 Derived Mesendoderm Cultured Cells	REMC
ChromHMM	chr1:22926200..22976400	Weak Repressed PolyComb	Blood & T-cell	Primary T helper naive cells fromÃ,Â peripheralÃ,Â blood	REMC
ChromHMM	chr1:22906200..22973000	Quiescent/Low	ENCODE	GM12878 Lymphoblastoid Cell Line	REMC
ChromHMM	chr1:22949200..23011000	Weak Repressed PolyComb	ENCODE	HMEC Mammary Epithelial Primary Cells	REMC
ChromHMM	chr1:22927600..22973800	Weak Repressed PolyComb	ENCODE	K562 Leukemia Cell Line	REMC
ChromHMM	chr1:22945600..22973400	Weak Repressed PolyComb	Blood & T-cell	Primary T CD8+ naive cells from peripheral blood	REMC
ChromHMM	chr1:22946000..22999400	Quiescent/Low	ES-deriv	H1 Derived Neuronal Progenitor Cultured Cells	REMC
ChromHMM	chr1:22945200..22980800	Weak Repressed PolyComb	ENCODE	HUVEC Umbilical Vein Endothelial Primary Cells	REMC
ChromHMM	chr1:22952600..23020000	Weak Repressed PolyComb	Blood & T-cell	Primary T helper cells fromÃ,Â peripheralÃ,Â blood	REMC
ChromHMM	chr1:22953200..22976000	Weak Repressed PolyComb	HSC & B-cell	Primary neutrophils fromÃ,Â peripheralÃ,Â blood	REMC
ChromHMM	chr1:22953400..22981200	Weak Repressed PolyComb	Sm. Muscle	Colon Smooth Muscle	REMC
ChromHMM	chr1:22953400..22983800	Quiescent/Low	Other	Pancreatic Islets	REMC
ChromHMM	chr1:22953600..22977200	Weak Repressed PolyComb	Blood & T-cell	Primary mononuclear cells fromÃ,Â peripheralÃ,Â blood	REMC

ChromHMM	chr1:22955000..23010000	Weak Repressed PolyComb	HSC & B-cell	Primary hematopoietic stem cells short term culture	REMC
ChromHMM	chr1:22954400..22974000	Weak Repressed PolyComb	Heart	Right Ventricle	REMC
ChromHMM	chr1:22954800..22999800	Weak Repressed PolyComb	Digestive	Stomach Mucosa	REMC
ChromHMM	chr1:22952600..22980600	Weak Repressed PolyComb	ENCODE	NHDF-Ad Adult Dermal Fibroblast Primary Cells	REMC
ChromHMM	chr1:22955800..22981800	Weak Repressed PolyComb	Epithelial	Foreskin Keratinocyte Primary Cells skin03	REMC
ChromHMM	chr1:22956200..22980000	Quiescent/Low	Other	Ovary	REMC
ChromHMM	chr1:22958400..22976400	Weak Repressed PolyComb	Other	Fetal Lung	REMC
ChromHMM	chr1:22961800..22973000	Quiescent/Low	Heart	Aorta	REMC
ChromHMM	chr1:22962000..22971600	Quiescent/Low	Digestive	Gastric	REMC
ChromHMM	chr1:22955800..22976400	Quiescent/Low	Muscle	Psoas Muscle	REMC
ChromHMM	chr1:22958400..22982400	Weak transcription	Heart	Right Atrium	REMC
ChromHMM	chr1:22956000..22993200	Quiescent/Low	Thymus	Thymus	REMC
ChromHMM	chr1:22964200..22973800	Weak Repressed PolyComb	ENCODE	HeLa-S3 Cervical Carcinoma Cell Line	REMC
ChromHMM	chr1:22963400..22979000	Weak Repressed PolyComb	ENCODE	HSMM Skeletal Muscle Myoblasts Cell Line	REMC
ChromHMM	chr1:22960800..22994400	Weak Repressed PolyComb	ENCODE	NHEK-Epidermal Keratinocyte Primary Cells	REMC
ChromHMM	chr1:22960800..22977400	Weak Repressed PolyComb	ENCODE	NHLF Lung Fibroblast Primary Cells	REMC
ChromHMM	chr1:22961200..22975400	Weak Repressed PolyComb	ENCODE	Osteoblast Primary Cells	REMC
ChromHMM	chr1:22955800..23035800	Weak Repressed PolyComb	Digestive	Small Intestine	REMC
ChromHMM	chr1:22957200..22979800	Weak Repressed PolyComb	Epithelial	Foreskin Keratinocyte Primary Cells skin02	REMC
ChromHMM	chr1:22958600..22997800	Weak Repressed PolyComb	HSC & B-cell	Primary hematopoietic stem cells	REMC
ChromHMM	chr1:22959800..22993200	Quiescent/Low	Blood & T-cell	Primary T CD8+ memory cells from peripheral blood	REMC
ChromHMM	chr1:22961400..22994200	Quiescent/Low	HSC & B-cell	Primary B cells from cord blood	REMC
ChromHMM	chr1:22962000..22982000	Quiescent/Low	ES-deriv	hESC Derived CD56+ Ectoderm Cultured Cells	REMC
ChromHMM	chr1:22962000..22997000	Quiescent/Low	ESC	H9 Cell Line	REMC
ChromHMM	chr1:22962400..22982000	Weak Repressed PolyComb	Neurosph	Ganglion Eminence derived primary cultured neurospheres	REMC
ChromHMM	chr1:22962600..22995600	Quiescent/Low	Blood & T-cell	Primary T helper memory cells from peripheral blood 1	REMC
ChromHMM	chr1:22966000..23067800	Weak Repressed PolyComb	ENCODE	Dnd41 TCell Leukemia Cell Line	REMC
ChromHMM	chr1:22969000..23035600	Weak Repressed PolyComb	Blood & T-cell	Primary T regulatory cells fromÃ,Â peripheralÃ,Â blood	REMC
ChromHMM	chr1:22971400..23030800	Quiescent/Low	Blood & T-cell	Primary T helper memory cells from peripheral blood 2	REMC

ChromHMM	chr1:22964000..22971600	Weak Repressed PolyComb	Myosat	Muscle Satellite Cultured Cells	REMC
ChromHMM	chr1:22964000..22973200	Weak Repressed PolyComb	Mesench	Bone Marrow Derived Cultured Mesenchymal Stem Cells	REMC
ChromHMM	chr1:22970400..22972200	Active TSS	Digestive	Rectal Mucosa Donor 31	REMC
ChromHMM	chr1:22971000..22972000	Repressed PolyComb	Muscle	Skeletal Muscle Male	REMC
ChromHMM	chr1:22971400..22972400	Repressed PolyComb	Sm. Muscle	Stomach Smooth Muscle	REMC
ChromHMM	chr1:22963400..22972800	Weak Repressed PolyComb	ENCODE	HSMM cell derived Skeletal Muscle Myotubes Cell Line	REMC
ChromHMM	chr1:22965000..22995800	Quiescent/Low	HSC & B-cell	Primary monocytes from peripheral blood	REMC
ChromHMM	chr1:22965600..22976200	Weak Repressed PolyComb	Other	Fetal Kidney	REMC
ChromHMM	chr1:22966000..22974000	Weak transcription	Other	Lung	REMC
ChromHMM	chr1:22966000..22999800	Quiescent/Low	ESC	HUES48 Cell Line	REMC
ChromHMM	chr1:22966200..22992400	Weak Repressed PolyComb	ESC	ES-WA7 Cell Line	REMC
ChromHMM	chr1:22966200..22999600	Quiescent/Low	iPSC	iPS-18 Cell Line	REMC
ChromHMM	chr1:22966600..22978600	Quiescent/Low	ES-deriv	hESC Derived CD56+ Mesoderm Cultured Cells	REMC
ChromHMM	chr1:22968400..22992400	Quiescent/Low	iPSC	iPS DF 6.9 Cell Line	REMC
ChromHMM	chr1:22968400..22999600	Quiescent/Low	iPSC	iPS-20b Cell Line	REMC
ChromHMM	chr1:22970800..23004600	Quiescent/Low	Other	Pancreas	REMC
ChromHMM	chr1:22968800..23003200	Weak Repressed PolyComb	Digestive	Sigmoid Colon	REMC
ChromHMM	chr1:22968000..22973400	Weak transcription	Other	Spleen	REMC
ChromHMM	chr1:22966000..22979000	Quiescent/Low	ENCODE	Monocytes-CD14+ RO01746 Primary Cells	REMC
ChromHMM	chr1:22965400..22979200	Weak Repressed PolyComb	ENCODE	NH-A Astrocytes Primary Cells	REMC
ChromHMM	chr1:22968600..22971600	Weak Repressed PolyComb	Brain	Brain Inferior Temporal Lobe	REMC
ChromHMM	chr1:22968600..22971600	Repressed PolyComb	Digestive	Fetal Stomach	REMC
ChromHMM	chr1:22968600..22972200	Weak transcription	ES-deriv	H1 BMP4 Derived Trophoblast Cultured Cells	REMC
ChromHMM	chr1:22968600..22972600	Quiescent/Low	ESC	H1 Cell Line	REMC
ChromHMM	chr1:22968600..22972600	Repressed PolyComb	IMR90	IMR90 fetal lung fibroblasts Cell Line	REMC
ChromHMM	chr1:22968600..22972600	Active TSS	Brain	Brain Hippocampus Middle	REMC
ChromHMM	chr1:22968600..22975200	Quiescent/Low	ESC	ES-UCSF4 Cell Line	REMC
ChromHMM	chr1:22968600..22976200	Weak Repressed PolyComb	Brain	Fetal Brain Male	REMC
ChromHMM	chr1:22968600..22978200	Weak Repressed PolyComb	Mesench	Mesenchymal Stem Cell Derived Chondrocyte Cultured Cells	REMC
ChromHMM	chr1:22968600..22978400	Quiescent/Low	Heart	Fetal Heart	REMC
ChromHMM	chr1:22968600..22979200	Quiescent/Low	Brain	Brain Germinal Matrix	REMC
ChromHMM	chr1:22968600..22979600	Quiescent/Low	ENCODE	A549 EtOH 0.02pct Lung Carcinoma Cell Line	REMC
ChromHMM	chr1:22968600..22979800	Quiescent/Low	Brain	Fetal Brain Female	REMC

ChromHMM	chr1:22968600..22981000	Weak Repressed PolyComb	HSC & B-cell	Primary hematopoietic stem cells G-CSF-mobilized Female	REMC
ChromHMM	chr1:22968600..22985000	Weak Repressed PolyComb	ESC	HUES6 Cell Line	REMC
ChromHMM	chr1:22968600..22985600	Weak Repressed PolyComb	ES-deriv	hESC Derived CD184+ Endoderm Cultured Cells	REMC
ChromHMM	chr1:22968600..22992800	Weak Repressed PolyComb	ESC	ES-I3 Cell Line	REMC
ChromHMM	chr1:22968600..22999800	Quiescent/Low	ESC	HUES64 Cell Line	REMC
ChromHMM	chr1:22968800..22972600	Repressed PolyComb	Blood & T-cell	Primary T cells fromÃ,Â peripheralÃ,Â blood	REMC
ChromHMM	chr1:22968800..22973200	Weak Repressed PolyComb	HSC & B-cell	Primary B cells from peripheral blood	REMC
ChromHMM	chr1:22968800..22978000	Quiescent/Low	Other	Placenta Amnion	REMC
ChromHMM	chr1:22969000..22971800	Weak Repressed PolyComb	Blood & T-cell	Primary T helper cells PMA-I stimulated	REMC
ChromHMM	chr1:22969000..22972600	Repressed PolyComb	Epithelial	Foreskin Fibroblast Primary Cells skin01	REMC
ChromHMM	chr1:22969000..22979200	Weak Repressed PolyComb	ES-deriv	H1 Derived Mesenchymal Stem Cells	REMC
ChromHMM	chr1:22969000..22979600	Weak Repressed PolyComb	Epithelial	Breast Myoepithelial Primary Cells	REMC
ChromHMM	chr1:22969000..22979600	Weak Repressed PolyComb	Neurosph	Cortex derived primary cultured neurospheres	REMC
ChromHMM	chr1:22969000..22999800	Quiescent/Low	HSC & B-cell	Primary Natural Killer cells fromÃ,Â peripheralÃ,Â blood	REMC
ChromHMM	chr1:22969200..22971600	Weak Repressed PolyComb	Mesench	Mesenchymal Stem Cell Derived Adipocyte Cultured Cells	REMC
ChromHMM	chr1:22969600..22972800	Repressed PolyComb	ES-deriv	H9 Derived Neuron Cultured Cells	REMC
ChromHMM	chr1:22969600..22974600	Weak Repressed PolyComb	Mesench	Adipose Derived Mesenchymal Stem Cell Cultured Cells	REMC
ChromHMM	chr1:22969800..22973600	Weak Repressed PolyComb	Epithelial	Breast variant Human Mammary Epithelial Cells (vHMEC)	REMC
ChromHMM	chr1:22969800..22979400	Weak Repressed PolyComb	HSC & B-cell	Primary hematopoietic stem cells G-CSF-mobilized Male	REMC
ChromHMM	chr1:22969800..22998200	Weak Repressed PolyComb	Blood & T-cell	Primary T cells from cord blood	REMC
ChromHMM	chr1:22970000..22971800	Active TSS	Adipose	Adipose Nuclei	REMC
ChromHMM	chr1:22970000..22971800	Weak Repressed PolyComb	Brain	Brain Angular Gyrus	REMC
ChromHMM	chr1:22970000..22973600	Weak Repressed PolyComb	Epithelial	Foreskin Melanocyte Primary Cells skin03	REMC
ChromHMM	chr1:22970000..22976000	Quiescent/Low	iPSC	iPS-15b Cell Line	REMC
ChromHMM	chr1:22970200..22972000	Active TSS	Digestive	Rectal Mucosa Donor 29	REMC
ChromHMM	chr1:22970200..22972600	Active TSS	Brain	Brain Anterior Caudate	REMC
ChromHMM	chr1:22970200..22972600	Active TSS	Brain	Brain Dorsolateral Prefrontal Cortex	REMC
ChromHMM	chr1:22970200..22972600	Bivalent/Poised TSS	Other	Fetal Adrenal Gland	REMC
ChromHMM	chr1:22970200..22972800	Active TSS	Brain	Brain Substantia Nigra	REMC
ChromHMM	chr1:22970200..22973000	Active TSS	Other	Placenta	REMC
ChromHMM	chr1:22970200..22999800	Weak Repressed PolyComb	Blood & T-cell	Primary T helper 17 cells PMA-I stimulated	REMC
ChromHMM	chr1:22970400..22972200	Active TSS	Muscle	Fetal Muscle Leg	REMC
ChromHMM	chr1:22970400..22972400	Repressed PolyComb	Epithelial	Foreskin Melanocyte Primary Cells skin01	REMC

ChromHMM	chr1:22970400..22972600	Active TSS	Digestive	Duodenum Mucosa	REMC
ChromHMM	chr1:22970600..22971600	Active TSS	Digestive	Colonic Mucosa	REMC
ChromHMM	chr1:22970600..22979000	Quiescent/Low	Heart	Left Ventricle	REMC
ChromHMM	chr1:22970800..22972000	Weak transcription	iPSC	iPS DF 19.11 Cell Line	REMC
ChromHMM	chr1:22970800..22975800	Repressed PolyComb	ENCODE	HepG2 Hepatocellular Carcinoma Cell Line	REMC
ChromHMM	chr1:22970800..22978400	Weak Repressed PolyComb	Muscle	Skeletal Muscle Female	REMC
ChromHMM	chr1:22971000..22972600	Active TSS	Sm. Muscle	Duodenum Smooth Muscle	REMC
ChromHMM	chr1:22971000..22972800	Repressed PolyComb	Epithelial	Foreskin Fibroblast Primary Cells skin02	REMC
ChromHMM	chr1:22971000..22974000	Weak transcription	Digestive	Esophagus	REMC
ChromHMM	chr1:22971400..22971600	Bivalent/Poised TSS	Sm. Muscle	Rectal Smooth Muscle	REMC
ChromHMM	chr1:22971200..22972000	Active TSS	Other	Liver	REMC
ChromHMM	chr1:22971200..22974200	Repressed PolyComb	ES-deriv	H9 Derived Neuronal Progenitor Cultured Cells	REMC
ChromHMM	chr1:22971200..22980200	Quiescent/Low	Digestive	Fetal Intestine Small	REMC
ChromHMM	chr1:22971200..22987400	Weak Repressed PolyComb	Digestive	Fetal Intestine Large	REMC
ChromHMM	chr1:22971400..22972200	Repressed PolyComb	Muscle	Fetal Muscle Trunk	REMC
ChromHMM	chr1:22971400..22972800	Active TSS	Brain	Brain Cingulate Gyrus	REMC

D. rs291985

A) Chromatin structure				
**Method**	**Location**	**Cell Type**	**Additional Info**	**Reference**
DNase-seq	chr1:22980684..22980952	Panislets		ENCODE

B) Histone modifications					
**Method**	**Location**	**Chromatin State**	**Tissue Group**	**Tissue**	**Reference**
ChromHMM	chr1:22891000..23030200	Quiescent/Low	Blood & T-cell	Primary T helper naive cells from peripheral blood	REMC
ChromHMM	chr1:22920200..23034000	Quiescent/Low	ES-deriv	H1 BMP4 Derived Mesendoderm Cultured Cells	REMC
ChromHMM	chr1:22949200..23011000	Weak Repressed PolyComb	ENCODE	HMEC Mammary Epithelial Primary Cells	REMC
ChromHMM	chr1:22946000..22999400	Quiescent/Low	ES-deriv	H1 Derived Neuronal Progenitor Cultured Cells	REMC
ChromHMM	chr1:22952600..23020000	Weak Repressed PolyComb	Blood & T-cell	Primary T helper cells fromÃ,Â peripheralÃ,Â blood	REMC
ChromHMM	chr1:22953400..22981200	Weak Repressed PolyComb	Sm. Muscle	Colon Smooth Muscle	REMC
ChromHMM	chr1:22953400..22983800	Quiescent/Low	Other	Pancreatic Islets	REMC
ChromHMM	chr1:22955000..23010000	Weak Repressed PolyComb	HSC & B-cell	Primary hematopoietic stem cells short term culture	REMC
ChromHMM	chr1:22954800..22999800	Weak Repressed PolyComb	Digestive	Stomach Mucosa	REMC
ChromHMM	chr1:22973000..23016800	Weak Repressed PolyComb	ENCODE	GM12878 Lymphoblastoid Cell Line	REMC

ChromHMM	chr1:22974400..23017400	Weak Repressed PolyComb	ENCODE	K562 Leukemia Cell Line	REMC
ChromHMM	chr1:22955800..22981800	Weak Repressed PolyComb	Epithelial	Foreskin Keratinocyte Primary Cells skin03	REMC
ChromHMM	chr1:22980400..23016000	Weak Repressed PolyComb	Brain	Brain Dorsolateral Prefrontal Cortex	REMC
ChromHMM	chr1:22958400..22982400	Weak transcription	Heart	Right Atrium	REMC
ChromHMM	chr1:22956000..22993200	Quiescent/Low	Thymus	Thymus	REMC
ChromHMM	chr1:22960800..22994400	Weak Repressed PolyComb	ENCODE	NHEK-Epidermal Keratinocyte Primary Cells	REMC
ChromHMM	chr1:22955800..23035800	Weak Repressed PolyComb	Digestive	Small Intestine	REMC
ChromHMM	chr1:22958600..22997800	Weak Repressed PolyComb	HSC & B-cell	Primary hematopoietic stem cells	REMC
ChromHMM	chr1:22959800..22993200	Quiescent/Low	Blood & T-cell	Primary T CD8+ memory cells from peripheral blood	REMC
ChromHMM	chr1:22961400..22994200	Quiescent/Low	HSC & B-cell	Primary B cells from cord blood	REMC
ChromHMM	chr1:22962000..22982000	Quiescent/Low	ES-deriv	hESC Derived CD56+ Ectoderm Cultured Cells	REMC
ChromHMM	chr1:22962000..22997000	Quiescent/Low	ESC	H9 Cell Line	REMC
ChromHMM	chr1:22962400..22982000	Weak Repressed PolyComb	Neurosph	Ganglion Eminence derived primary cultured neurospheres	REMC
ChromHMM	chr1:22962600..22995600	Quiescent/Low	Blood & T-cell	Primary T helper memory cells from peripheral blood 1	REMC
ChromHMM	chr1:22966000..23067800	Weak Repressed PolyComb	ENCODE	Dnd41 TCell Leukemia Cell Line	REMC
ChromHMM	chr1:22969000..23035600	Weak Repressed PolyComb	Blood & T-cell	Primary T regulatory cells fromÃ,Â peripheralÃ,Â blood	REMC
ChromHMM	chr1:22971400..23030800	Quiescent/Low	Blood & T-cell	Primary T helper memory cells from peripheral blood 2	REMC
ChromHMM	chr1:22976400..23028800	Quiescent/Low	Blood & T-cell	Primary T helper naive cells fromÃ,Â peripheralÃ,Â blood	REMC
ChromHMM	chr1:22965000..22995800	Quiescent/Low	HSC & B-cell	Primary monocytes from peripheral blood	REMC
ChromHMM	chr1:22966000..22999800	Quiescent/Low	ESC	HUES48 Cell Line	REMC
ChromHMM	chr1:22966200..22992400	Weak Repressed PolyComb	ESC	ES-WA7 Cell Line	REMC
ChromHMM	chr1:22966200..22999600	Quiescent/Low	iPSC	iPS-18 Cell Line	REMC
ChromHMM	chr1:22968400..22992400	Quiescent/Low	iPSC	iPS DF 6.9 Cell Line	REMC
ChromHMM	chr1:22968400..22999600	Quiescent/Low	iPSC	iPS-20b Cell Line	REMC
ChromHMM	chr1:22970800..23004600	Quiescent/Low	Other	Pancreas	REMC
ChromHMM	chr1:22972200..23005000	Quiescent/Low	Digestive	Gastric	REMC
ChromHMM	chr1:22974200..23010600	Weak Repressed PolyComb	Epithelial	Breast variant Human Mammary Epithelial Cells (vHMEC)	REMC
ChromHMM	chr1:22978000..23006000	Weak Repressed PolyComb	Other	Placenta Amnion	REMC

ChromHMM	chr1:22968800..23003200	Weak Repressed PolyComb	Digestive	Sigmoid Colon	REMC
ChromHMM	chr1:22968600..22981000	Weak Repressed PolyComb	HSC & B-cell	Primary hematopoietic stem cells G-CSF-mobilized Female	REMC
ChromHMM	chr1:22968600..22985000	Weak Repressed PolyComb	ESC	HUES6 Cell Line	REMC
ChromHMM	chr1:22968600..22985600	Weak Repressed PolyComb	ES-deriv	hESC Derived CD184+ Endoderm Cultured Cells	REMC
ChromHMM	chr1:22968600..22992800	Weak Repressed PolyComb	ESC	ES-I3 Cell Line	REMC
ChromHMM	chr1:22968600..22999800	Quiescent/Low	ESC	HUES64 Cell Line	REMC
ChromHMM	chr1:22969000..22999800	Quiescent/Low	HSC & B-cell	Primary Natural Killer cells fromÃ,Â peripheralÃ,Â blood	REMC
ChromHMM	chr1:22969800..22998200	Weak Repressed PolyComb	Blood & T-cell	Primary T cells from cord blood	REMC
ChromHMM	chr1:22970200..22999800	Weak Repressed PolyComb	Blood & T-cell	Primary T helper 17 cells PMA-I stimulated	REMC
ChromHMM	chr1:22974000..22992600	Weak transcription	Other	Spleen	REMC
ChromHMM	chr1:22974200..22997000	Weak Repressed PolyComb	Heart	Right Ventricle	REMC
ChromHMM	chr1:22971200..22987400	Weak Repressed PolyComb	Digestive	Fetal Intestine Large	REMC
ChromHMM	chr1:22971800..22992000	Quiescent/Low	Blood & T-cell	Primary T helper cells PMA-I stimulated	REMC
ChromHMM	chr1:22973000..22985200	Weak Repressed PolyComb	Heart	Aorta	REMC
ChromHMM	chr1:22977400..22985600	Repressed PolyComb	ENCODE	NHLF Lung Fibroblast Primary Cells	REMC
ChromHMM	chr1:22972600..23000000	Weak Repressed PolyComb	ESC	H1 Cell Line	REMC
ChromHMM	chr1:22973200..22992000	Quiescent/Low	HSC & B-cell	Primary B cells from peripheral blood	REMC
ChromHMM	chr1:22973400..22998000	Quiescent/Low	Blood & T-cell	Primary T CD8+ naive cells from peripheral blood	REMC
ChromHMM	chr1:22973600..23002400	Repressed PolyComb	IMR90	IMR90 fetal lung fibroblasts Cell Line	REMC
ChromHMM	chr1:22974400..22981000	Weak Repressed PolyComb	Mesench	Mesenchymal Stem Cell Derived Adipocyte Cultured Cells	REMC
ChromHMM	chr1:22974400..23005000	Quiescent/Low	ES-deriv	H1 BMP4 Derived Trophoblast Cultured Cells	REMC
ChromHMM	chr1:22975200..22985400	Weak Repressed PolyComb	ESC	ES-UCSF4 Cell Line	REMC
ChromHMM	chr1:22977000..23004000	Quiescent/Low	iPSC	iPS-15b Cell Line	REMC
ChromHMM	chr1:22978400..23005000	Weak Repressed PolyComb	Heart	Fetal Heart	REMC
ChromHMM	chr1:22975800..22981800	Weak Repressed PolyComb	ENCODE	HeLa-S3 Cervical Carcinoma Cell Line	REMC
ChromHMM	chr1:22976000..22984200	Weak Repressed PolyComb	Digestive	Esophagus	REMC
ChromHMM	chr1:22976600..22982800	Weak Repressed PolyComb	Other	Fetal Lung	REMC
ChromHMM	chr1:22976800..22981200	Weak Repressed PolyComb	Other	Fetal Kidney	REMC
ChromHMM	chr1:22976800..22983000	Weak Repressed PolyComb	Brain	Fetal Brain Male	REMC
ChromHMM	chr1:22976000..22996200	Quiescent/Low	HSC & B-cell	Primary neutrophils fromÃ,Â peripheral blood	REMC

ChromHMM	chr1:22976400..22993800	Weak Repressed PolyComb	Muscle	Psoas Muscle	REMC
ChromHMM	chr1:22977000..22989200	Quiescent/Low	Blood & T-cell	Primary T cells effector/memory enriched from peripheral blood	REMC
ChromHMM	chr1:22977200..22988400	Quiescent/Low	Blood & T-cell	Primary mononuclear cells fromÃ,Â peripheralÃ,Â blood	REMC
ChromHMM	chr1:22978400..23000200	Repressed PolyComb	Muscle	Skeletal Muscle Male	REMC
ChromHMM	chr1:22978600..23000200	Weak Repressed PolyComb	ES-deriv	hESC Derived CD56+ Mesoderm Cultured Cells	REMC
ChromHMM	chr1:22979600..23000200	Repressed PolyComb	Mesench	Adipose Derived Mesenchymal Stem Cell Cultured Cells	REMC
ChromHMM	chr1:22980200..22997800	Quiescent/Low	Thymus	Fetal Thymus	REMC
ChromHMM	chr1:22980800..23000200	Weak Repressed PolyComb	ENCODE	HepG2 Hepatocellular Carcinoma Cell Line	REMC
ChromHMM	chr1:22979000..22981400	Repressed PolyComb	ENCODE	HSMM Skeletal Muscle Myoblasts Cell Line	REMC
ChromHMM	chr1:22978200..22985000	Repressed PolyComb	Myosat	Muscle Satellite Cultured Cells	REMC
ChromHMM	chr1:22978200..22992000	Repressed PolyComb	Mesench	Mesenchymal Stem Cell Derived Chondrocyte Cultured Cells	REMC
ChromHMM	chr1:22978400..22983600	Repressed PolyComb	Epithelial	Foreskin Fibroblast Primary Cells skin01	REMC
ChromHMM	chr1:22978600..22981000	Repressed PolyComb	Blood & T-cell	Primary T cells fromÃ,Â peripheralÃ,Â blood	REMC
ChromHMM	chr1:22978600..22991200	Repressed PolyComb	Mesench	Bone Marrow Derived Cultured Mesenchymal Stem Cells	REMC
ChromHMM	chr1:22979200..22988400	Repressed PolyComb	ES-deriv	H1 Derived Mesenchymal Stem Cells	REMC
ChromHMM	chr1:22979400..22984800	Repressed PolyComb	HSC & B-cell	Primary hematopoietic stem cells G-CSF-mobilized Male	REMC
ChromHMM	chr1:22979600..22983800	Repressed PolyComb	Neurosph	Cortex derived primary cultured neurospheres	REMC
ChromHMM	chr1:22980200..22981200	Weak Repressed PolyComb	Epithelial	Breast Myoepithelial Primary Cells	REMC
ChromHMM	chr1:22980600..22986400	Weak Repressed PolyComb	ES-deriv	H9 Derived Neuronal Progenitor Cultured Cells	REMC
ChromHMM	chr1:22980800..22984400	Weak Repressed PolyComb	ES-deriv	H9 Derived Neuron Cultured Cells	REMC
ChromHMM	chr1:22980800..22988800	Quiescent/Low	iPSC	iPS DF 19.11 Cell Line	REMC
ChromHMM	chr1:22979200..22990000	Repressed PolyComb	Epithelial	Foreskin Fibroblast Primary Cells skin02	REMC
ChromHMM	chr1:22979800..22982400	Repressed PolyComb	Epithelial	Foreskin Keratinocyte Primary Cells skin02	REMC
ChromHMM	chr1:22980000..22982000	Repressed PolyComb	Epithelial	Foreskin Melanocyte Primary Cells skin01	REMC
ChromHMM	chr1:22978800..22985600	Repressed PolyComb	ENCODE	HSMM cell derived Skeletal Muscle Myotubes Cell Line	REMC
ChromHMM	chr1:22979000..22987400	Weak Repressed PolyComb	ENCODE	Monocytes-CD14+ RO01746 Primary Cells	REMC
ChromHMM	chr1:22979200..22981000	Repressed PolyComb	Digestive	Fetal Stomach	REMC
ChromHMM	chr1:22979200..22985200	Repressed PolyComb	ENCODE	NH-A Astrocytes Primary Cells	REMC
ChromHMM	chr1:22979200..22987400	Weak Repressed PolyComb	Brain	Brain Germinal Matrix	REMC
ChromHMM	chr1:22979800..22992400	Weak Repressed PolyComb	Brain	Fetal Brain Female	REMC
ChromHMM	chr1:22980000..22987000	Weak Repressed PolyComb	Other	Ovary	REMC
ChromHMM	chr1:22980600..22985400	Weak Repressed PolyComb	Sm. Muscle	Rectal Smooth Muscle	REMC
ChromHMM	chr1:22980800..22994200	Quiescent/Low	Other	Lung	REMC

ChromHMM	chr1:22979600..22981600	Bivalent/Poised TSS	Sm. Muscle	Stomach Smooth Muscle	REMC
ChromHMM	chr1:22979600..22982200	Active TSS	Other	Placenta	REMC
ChromHMM	chr1:22979600..22982800	Active TSS	Adipose	Adipose Nuclei	REMC
ChromHMM	chr1:22979600..22983000	Repressed PolyComb	ENCODE	Osteoblast Primary Cells	REMC
ChromHMM	chr1:22979600..22983200	Weak Repressed PolyComb	ENCODE	A549 EtOH 0.02pct Lung Carcinoma Cell Line	REMC
ChromHMM	chr1:22979800..22981000	Bivalent/Poised TSS	Muscle	Skeletal Muscle Female	REMC
ChromHMM	chr1:22979800..22981200	Active TSS	Digestive	Colonic Mucosa	REMC
ChromHMM	chr1:22979800..22981200	Active TSS	Heart	Left Ventricle	REMC
ChromHMM	chr1:22979800..22981200	Active TSS	Digestive	Rectal Mucosa Donor 31	REMC
ChromHMM	chr1:22979800..22981800	Active TSS	Sm. Muscle	Duodenum Smooth Muscle	REMC
ChromHMM	chr1:22979800..22982200	Bivalent/Poised TSS	Muscle	Fetal Muscle Leg	REMC
ChromHMM	chr1:22979800..22982400	Repressed PolyComb	Epithelial	Foreskin Melanocyte Primary Cells skin03	REMC
ChromHMM	chr1:22979800..22982600	Bivalent/Poised TSS	Other	Fetal Adrenal Gland	REMC
ChromHMM	chr1:22979800..22982600	Active TSS	Brain	Brain Substantia Nigra	REMC
ChromHMM	chr1:22979800..22982600	Active TSS	Brain	Brain Hippocampus Middle	REMC
ChromHMM	chr1:22979800..22983600	Active TSS	Brain	Brain Anterior Caudate	REMC
ChromHMM	chr1:22980000..22981600	Active TSS	Brain	Brain Inferior Temporal Lobe	REMC
ChromHMM	chr1:22980000..22982400	Active TSS	Brain	Brain Cingulate Gyrus	REMC
ChromHMM	chr1:22980400..22981400	Bivalent/Poised TSS	Digestive	Rectal Mucosa Donor 29	REMC
ChromHMM	chr1:22980600..22983400	Repressed PolyComb	ENCODE	NHDF-Ad Adult Dermal Fibroblast Primary Cells	REMC
ChromHMM	chr1:22980800..22982400	Weak Repressed PolyComb	Muscle	Fetal Muscle Trunk	REMC
ChromHMM	chr1:22980800..22982600	Repressed PolyComb	ENCODE	HUVEC Umbilical Vein Endothelial Primary Cells	REMC
ChromHMM	chr1:22980600..22981000	Bivalent/Poised TSS	Digestive	Duodenum Mucosa	REMC
ChromHMM	chr1:22980600..22981200	Repressed PolyComb	Digestive	Fetal Intestine Small	REMC
ChromHMM	chr1:22980600..22981400	Active TSS	Brain	Brain Angular Gyrus	REMC
ChromHMM	chr1:22980800..22981000	Active TSS	Other	Liver	REMC
